# MnoSR removal in *Mycobacterium smegmatis* triggers broad transcriptional response to 1,3-propanediol and glucose as sole carbon sources

**DOI:** 10.3389/fcimb.2024.1427829

**Published:** 2024-07-24

**Authors:** Renata Płocińska, Katarzyna Struś, Małgorzata Korycka-Machała, Przemysław Płociński, Magdalena Kuzioła, Anna Żaczek, Marcin Słomka, Jarosław Dziadek

**Affiliations:** ^1^ Institute of Medical Biology of the Polish Academy of Sciences, Łódź, Poland; ^2^ Department of Immunology and Infectious Biology, Faculty of Biology and Environmental Protection, University of Łódz, Łódź, Poland; ^3^ BioMedChem Doctoral School of the UL and Łódź Institutes of the Polish Academy of Sciences, Łódź, Poland; ^4^ Department of Microbiology, College of Medical Sciences, University of Rzeszów, Rzeszów, Poland; ^5^ Biobank Lab, Department of Molecular Biophysics, Faculty of Biology and Environmental Protection, University of Łódź, Łódź, Poland

**Keywords:** histidine sensor kinase MnoS, response regulator MnoR, mycobacterium smegmatis, methylotrophic metabolism, RNA-Seq

## Abstract

**Introduction:**

The two-component signal transduction systems play an essential role in the adaptation of bacteria to changing environmental conditions. One of them is the MnoSR system involved in the regulation of methylotrophic metabolism in M. smegmatis.

**Methods:**

Mycobacterium smegmatis mutant strains ΔmnoS, ΔmnoR and ΔmnoS/R lacking functional mnoS, mnoR and both genes were generated using a homologous recombination approach. MnoR recombinant protein was purified by affinity column chromatography. The present study employs molecular biology techniques: cloning strategies, global RNA sequencing, qRT-PCR, EMSA, Microscale thermophoresis, and bioinformatics analysis.

**Results and discussion:**

The ∆mnoS, ∆mnoR, and ∆mnoS/R mutant strains were generated and cultured in the presence of defined carbon sources. Growth curve analysis confirmed that inactivation of the MnoSR impairs the ability of M. smegmatis cells to use alcohols such as 1,3-propanediol and ethanol but improves the bacterial growth on ethylene glycol, xylitol, and glycerol. The total RNA sequencing method was employed to understand the importance of MnoSR in the global responses of mycobacteria to limited carbon access and in carbon-rich conditions. The loss of MnoSR significantly affected carbon utilization in the case of mycobacteria cultured on glucose or 1,3-propanediol as sole carbon sources as it influenced the expression of multiple metabolic pathways. The numerous transcriptional changes could not be linked to the presence of evident MnoR DNA-binding sites within the promotor regions for the genes outside of the mno operon. This was confirmed by EMSA and microscale thermophoresis with mutated MnoR binding consensus region. Our comprehensive analysis highlights the system’s vital role in metabolic adaptability, providing insights into its potential impact on the environmental survival of mycobacteria.

## Introduction

The genus *Mycobacterium* consists of pathogenic species belonging to the *Mycobacterium tuberculosis* complex, which include mycobacteria capable of causing tuberculosis in humans (*Mycobacterium tuberculosis*) and animals (*Mycobacterium bovis*), non-tuberculous, atypical mycobacteria causing mycobacteriosis, which includes, among others, *Mycobacterium avium complex*, *Mycobacterium abscessus*, *Mycobacterium marinum* and mycobacteria causing leprosy – *Mycobacterium leprae*. *Mycobacterium smegmatis* (*M. smegmatis*) is a non-pathogenic and fast-growing species commonly used for research analysis in laboratory experiments. Members of the Mycobacteriaceae family are known to adapt to different environmental niches, utilizing various sources of carbon, oxygen and nitrogen. Carbon is a chemical element necessary for the proper growth of bacteria and an important component of biological macromolecules. Therefore, the cell’s response in carbon-limited conditions is crucial for bacterial survival and involves the interplay of signaling pathways. The methylotrophy metabolism allows microorganisms to utilize single-carbon (C_1_) compounds as the sole sources of carbon and energy. Methanol is one of the simplest organic molecules and the crucial carbon source for methylotrophic metabolism in bacteria (Anthony, 1991). It is a widely available, natural product of plant metabolism emitted to the atmosphere (Fall and Benson, 1996). The methanol dehydrogenase (MDH) is a key enzyme in methanol metabolism, it has the ability to catalyze the oxidation of methanol to formaldehyde. Methanol dehydrogenases may differ from each other among bacteria. The electron microscopic analyses of purified N,N’ -dimethyl-4-nitrosoaniline (NDMA) oxidoreductases (MNOs) from the gram-positive methylotrophic bacteria *Amycolatopsis methanolica* and *Mycobacterium gastri* MB19 revealed high degree identity in protein structures and significant identity to decameric methanol dehydrogenase of *Bacillus methanolicus* Cl (Bystrykh et al., 1993a). In addition to methanol oxidation, the formaldehyde dismutase activities of MDHs were reported in *A. methanolica* and *M. gastri* (Bystrykh et al., 1993b). Also, differences in structural and catalytic properties for NDMA-dependent alcohol dehydrogenase (NDMA-ADH) from *A. methanolica* were reported in comparison to methanol-oxidizing enzyme of *A. methanolica*, *Mycobacterium gastri* and *Bacillus methanolica C1* (Van Ophem et al., 1993). In Gram-negative methylotrophic bacteria, the NAD(P)-independent MDHs that contain pyrroloquinoline quinone (PQQ) as a prosthetic group were reported (Anthony, 2000).

The methylotrophic metabolism has been poorly described in mycobacteria. Tuberculosis bacteria have the ability to co-metabolize many carbon substrates inside host cells, and central carbon metabolism (CCM) plays a key role in their physiology and pathogenicity. Numerous studies have confirmed the presence in *M. tuberculosis* enzymes of the CCM pathway, including the tricarboxylic acid cycle, glycolysis, the pentose phosphate pathway, and their ability to use a number of glycolytic carbon substrates, including sugars and triglycerides (Xu et al., 2022). Several mycobacterial strains, such as *Mycobacterium flavescens*, *Mycobacterium gastri*, *Mycobacterium neoaurum*, *Mycobacterium parafortuitum*, *Mycobacterium peregrinum*, *Mycobacterium phlei*, *Mycobacterium smegmatis*, and *Mycobacterium vaccae*, were found to grow on carbon monoxide (CO) and methanol as the sole source of carbon and energy and exhibited ribulose-1,5-bisphosphate carboxylase/oxygenase (RuBisCO) activity. Interestingly, *M. tuberculosis* was not able to utilize methanol as the sole carbon and energy source and the RuBisCO activity was not detected, suggesting tuberculosis bacilli uses a different CO_2_ conversion pathway to organic material than the Calvin cycle. Moreover, the most common C_1_ assimilation pathways in procaryote-ribulose monophosphate (RuMP) and serine cycle are suggested to be absent in mycobacteria, and the cellular materials from CO and methanol have been synthesized through the eukaryote-specific Calvin cycle and xylulose monophosphate pathway (XuMP) (Park et al., 2003).

The facultatively chemolithotrophic *Mycobacterium* sp. strain JC1 has the ability to assimilate CO and methanol as a source of carbon and energy and utilizes both RuBisCO and XuMP pathways (Park et al., 2003). The *n,n-9-dimethyl-4-nitrosoaniline oxidoreductase* (*mdo*) deficient mutant of *Mycobacterium* sp. strain JC1 was not able to grow on methanol as the sole carbon source and was identified as a key enzyme for primary methanol oxidation (Park et al., 2010).

It was recently reported that *M. smegmatis* possesses an N,N-dimethyl-p-nitrosoaniline (NDMA)-dependent methanol dehydrogenase (Mno), which is necessary for methanol utilization by these bacteria (Dubey et al., 2018). qRT-PCR data revealed overexpression of *mno* in the presence of methanol and formaldehyde, whereas in the presence of glucose, the expression of *mno* was not affected. Another major formaldehyde dehydrogenase (MscR) in *M. smegmatis* has the ability to oxidize formaldehyde as well as methanol (Dubey et al., 2018). Growth kinetics analysis showed the *M. smegmatis* knock-out mutant cells lacking functional MscR were growing in the presence of glucose and methanol, but the growth was not detected in the presence of formaldehyde (Vargas et al., 2016). Interestingly, MscR cannot complement for the loss of Mno and Mno is unable to complement for the loss of MscR (Dubey et al., 2018). Dihydroxyacetone synthase (DHAS) is the key enzyme of the XuMP pathway reported in *Mycobacterium* JC1 (Park et al., 2003). MSMEG_3103 encoding the transketolase, which shares 60% sequence identity with DHAS from *Mycobacterium* JC1 was found to be downregulated during the growth of *M. smegmatis* cells in the presence of methanol and the knockout Δ*msmeg_3103* cells were able to utilize glycerol and methanol as the sole carbon source indicating the absence of the XuMP pathway in *M. smegmatis* (Dubey et al., 2018).

To effectively respond to changing environmental conditions, mycobacteria exploit two-component signal transduction systems (TCSSs). A typical two-component system is composed of a membrane-bound histidine sensor kinase (HK) that, upon detecting stress signals in the external environment, undergoes autophosphorylation on a conserved histidine residue. This is followed by a phosphotransfer to a unique aspartate residue on the regulatory domain of cytosolic response regulator (RR) by a phosphor-relay process, which activates the output domain of RR and promotes or represses gene transcription of selected genes (Hoch, 2000). Earlier studies reported the involvement of a two-component system regulating methanol oxidation in *Methylobacterium extorguens* and formaldehyde oxidation in *Paracoccus denitrificans* (Springer et al., 1998; Harms et al., 2001). A novel two-component system composed of a sensor kinase MnoS and response regulator MnoR, involved in methylotrophic metabolism regulation in *M. smegmatis* has been recently identified (Dubey and Jain, 2019). MnoS undergoes autophosphorylation and phosphorylates the conserved aspartate residue Asp60 of its cognate response regulator MnoR. It was reported the knockout mutant Δ*mnoSR* strain was unable to grow in the presence of methanol as the sole carbon source and MnoSR regulates Mno in a positive manner. MnoSR was found to regulate the expression of putative 1,3-propanediol dehydrogenase (MSMEG_6239), and the Δ*mnoSR* strain was unable to grow in the presence of 1,3-propanediol as the sole source of carbon. EMSA confirmed the binding of MnoR to the *mno* promoter region (Dubey and Jain, 2019). Here, we made an attempt to extend the study on the MnoSR two-component system and check whether it participates in the regulation of metabolic processes other than methylotrophic metabolism in *M. smegmatis* cells. For this purpose, we decided to perform a global transcriptomic analysis (RNA-seq) for the Δ*mnoSR* mutant under carbon starvation conditions and in the presence of either glucose or propanediol as the sole carbon source. Comparative analysis of transcriptomic data revealed large sets of genes whose expression changes depending on the availability of the carbon source, either directly or indirectly due to the presence of a functional MnoSR system.

## Materials and methods

### Strains and bacterial growth conditions

*E. coli* strains were grown in Luria-Bertani LB broth (LB) supplemented with 100 µg/mL ampicillin (Amp) and 50 µg/mL kanamycin (Kan), if required. *M. smegmatis* strains were cultured in Middlebrook 7H9 broth supplemented with 10% oleic acid-albumin-dextrose-catalase (OADC) and 0.05% Tween 80 and 25 µg/mL Kan, if required. Mycobacterial transformants were selected on Middlebrook 7H10 agar plates enriched with OADC and 0.2% glycerol. For growth experiments a defined carbon-free Sauton’s medium was applied (0.05% KH_2_PO_4_, 0.05% MgSO_4_, 0.0001% ZnSO_4_, 0.16% NH_4_Cl, 0.015% tyloxapol). For growth assays under nitrogen limitation a nitrogen-free Sauton’s medium was applied (0.05% KH_2_PO_4_, 0.05% MgSO_4_, 0.2% citric acid, 0.005% ferric citrate, 0.2% glycerol, 0.0001% ZnSO_4_, 0.015% tyloxapol).

### Gene cloning strategies

Standard molecular biology protocols were used for all cloning strategies (Sambrook and Russell, 2001). All PCR products were obtained using thermostable AccuPrime Pfx DNA polymerase (Invitrogen), cloned initially into a blunt vector (pJET1.2; Thermo Fisher), sequenced, and then released by digestion with appropriate restriction enzymes before ligation into the final vectors.

### Construction of gene replacement vectors and complementation plasmids

To perform unmarked deletions of *mnoS* (*msmeg_6238*), *mnoR* (*msmeg_6236*) and (*mnoS*, *mnoR*) (*msmeg_6236*, *msmeg_6238*) genes in *M. smegmatis*, we used a suicidal recombination delivery vector based on p2NIL (Parish and Stoker, 2000). The recombination vector carried the 5’end of *mnoS*, *mnoR*, *mnoS/R* with the upstream region connected to the 3’ end of the gene with the downstream region, amplified with the primers shown in **Supplementary Table S1** in the **Supplementary Materials**. Finally, the PacI screening cassette from pGOAL17 was inserted into the constructs, resulting in the suicide delivery vectors pKS10, pKS11, pKS12 carrying Δ*mnoS*, Δ*mnoR*, and Δ*mnoS*/*R* respectively; these were used to engineer the directed *M. smegmatis* mutants as described previously (Pawelczyk et al., 2011).

### Disruption of M. smegmatis mnoS and mnoR

The protocol of (Parish and Stoker, 2000) was used to disrupt the *M. smegmatis mnoS*, *mnoR*, *mnoS/R* genes at its native loci on the chromosome. The suicidal recombination plasmids DNA (pKS10, pKS11, pKS12) were treated with NaOH (0.2 mM) and integrated into the *M. smegmatis* chromosome by homologous recombination. The resulting single crossover recombinant (SCO) mutant colonies were blue, Kan^R^ and sensitive to sucrose. The site of recombination was confirmed by PCR and Southern hybridization. The SCO strains were further processed to select for double crossover (DCO) mutants that were white, Kan^S^ and resistant to sucrose (2%). The genotypes of obtained mutant DCO strains were confirmed by PCR and Southern hybridization using the Amersham ECL Direct Nucleic Acid Labelling And Detection System (GE Healthcare) following the manufacturer’s instructions. Primers used for PCR amplification are listed in **Supplementary Table S1**.

### Construction of complementation plasmids

*M. smegmatis* genes *mnoS*, *mnoR* and (*mnoS*/*R*), were amplified by PCR and cloned downstream from the P*_ami_* promoter. Mycobacterial genes were cloned first into the BamHI-XbaI sites of pJam2, and the cloned genes and P*_ami_* promoter were then excised with HindIII and XbaI and cloned into the integration vector pMV306Kan^R^. All plasmids and oligonucleotides used in this work are listed in **Supplementary Table S1** in the **Supplementary Materials**.

### Cloning, expression and purification of MnoR protein

*M. smegmatis mnoR* was amplified by PCR and cloned into pJet1.2 blunt vector, excised with BamHI/HindIII restriction enzymes and cloned into the final vector – pHIS resulting as pKS23 recombinant plasmid. The pKS23 plasmid DNA was transformed into *E. coli* BL21 DE3 cells. Single colonies were inoculated into 5 mL liquid medium, grown overnight, and diluted 100-fold into fresh medium (500 mL). After the colonies were grown to mid exponential phase (OD_600_ nm 0.5 to 0.6), protein expression was induced by the addition of IPTG (isopropyl β-D-1-thiogalactopyranoside) to 0.4 mM. After overnight incubation in 20°C, cells were harvested, sonicated, and centrifuged to separate the soluble and insoluble fractions. MnoR protein was purified from the soluble fraction by affinity column chromatography using cobalt resin and buffers containing 50 mM Tris-HCl pH 7.5, 1 M NaCl, 10 mM imidazole, 10% glycerol. The recombinant protein was eluted with 0.5 M imidazole, passed through PD-10 column Sephadex TMG-25M (GE Healthcare) and concentrated using concentrators with a 10,000 molecular-weight cut-off polyethersulfone (PES) membrane (Protein concentrator PES 10K MWCO, 5-20 mL, ThermoFisher Scientific).

### Growth assays under variable carbon sources

*M. smegmatis* strains Δ*mnoR*, Δ*mnoS*, Δ*mnoS*/*R* and wild-type were grown in 7H9 medium supplemented with Tween 80 and OADC up to the logarithmic stage of growth. Cells were centrifuged and washed three times with carbon-free Sauton’s medium, to achieve carbon starvation. Next, cells, diluted to OD_600_ 0.1 in the same medium and a tested carbon compound was added to each culture at a specific concentration (1,3-propanediol, ethanol, xylitol, ethylene glycol all at 2% final concentration; glycerol at 0.2%; fructose, maltose, lactose, mannose, ribose, xylose, galactose, sodium formate and sodium pyruvate, all at 0.5% final concentration). Cultures were carried out at a temperature of 37°C with shaking and the OD_600_ measurements were performed at 6, 9, 12, 24, 48 hours of the experiment. For some experiments tested strains grown in 7H9/OADC/Tween 80 were centrifuged, washed three times in carbon-free Sautons medium and diluted to OD_600_ 0.1 in the same medium supplemented with glucose 0.1%. Cultures were carried out at 37°C with shaking for 24h. Next, cells were centrifuged, washes three times in carbon-free Sauton’s medium and diluted to OD_600_ 0.1 in the same medium supplemented with xylitol 2%, glycerol 0.2% and ethylene glycol 2%. In order to monitor the kinetics of growth during lag phase of culture growth, the OD_600_ readings were performed at 1.5, 3, 4.5, 6, 7.5, 9, 12, 24, 48h of the experiment. Each experiment was conducted in three independent repetitions.

### Growth assays under variable nitrogen sources

*M. smegmatis* strains Δ*mnoR*, Δ*mnoS*, Δ*mnoS*/*R* and wild-type were grown in 7H9 medium supplemented with Tween 80 and OADC up to the logarithmic stage of growth. To induce nitrogen starvation, cells were washed three times in a nitrogen-free Sauton’s medium. The OD_600_ was adjusted to 0.2 in the same medium, and the cells were grown for 16 hours at 37 °C. Next, cells were harvested by centrifugation and diluted to OD_600_ 0.1 in Sauton’s medium supplemented with various substances that were tested as sole nitrogen sources: ammonium sulphate, urea (pH 4.5), uric acid, histidine (pH 9.5), leucine (pH 9.5), allantoin, hydantoin, proline (pH 9.5), methionine (pH 9.5), L-glutamic acid (all at 10 mM concentration), acetamide (5 mM). 7H9 medium containing OADC and Tween 80 was used as a positive control, and nitrogen-free Sauton’s medium was the negative control. The kinetics of the growth was monitored by measuring absorbance at 600 nm at 6, 9, 12, 24, 48  hours of growth. The experiment was repeated three times, independently.

### RNA isolation, removal of rRNA and library preparation

*M. smegmatis* strain lacking intact *mnoS/R* grown in Middlebrook 7H9 liquid medium supplemented with 2% glucose and 0.015% tyloxapol up to logarithmic phase of growth were centrifuged and washed three times in 7H9 medium. The experiment was conducted in three experimental variants. The OD_600_ was adjusted to 0.1 in 7H9 medium supplemented with 0.1% glucose for carbon starvation and 2% glucose or 0.1% glucose with 2% 1,3-propoanediol for carbon-rich conditions. The strains were propagated at 37 °C with shaking for another 16 h and centrifuged. Strains grown in rich medium were centrifuged when they reached an OD_600_ of approximately 0.8 - 1.0, strains grown under carbon starvation conditions were centrifuged when they stopped dividing (at approximately 1.5 OD_600_).

Total RNA was extracted using TRIzol reagent as described previously (Antczak et al., 2018) (Pawelczyk et al., 2011). Cells were disrupted twice using the MP disruptor system with the Quick prep adapter (MP Biomedicals) and 0.1 mm silica spheres (45 sec, 6.0 m/s with 5 min intervals on ice). To remove DNA contamination DNase I turbo (Invitrogen) was used according to manufacturer’s instructions. The RNA quantity and integrity was verified using an Agilent 2100 BioAnalyzer following the manufacturer’s protocol (Agilent RNA 600 Nano Kit). Next, the RNA samples were purified using AMPure magnetic beads (Becton Dickinson), the Ribo-Zero rRNA Removal Kit (Illumina) was applied in order to remove rRNA, and the libraries were prepared using KAPA Stranded RNA-seq Library Preparation Kit (KAPA Biosystems). The protocol included several stages: RNA fragmentation, cDNA synthesis, second strand synthesis, DNA end repair and adenylation, adapter ligation and library amplification. Then indexes were assigned to the obtained libraries, enabling their subsequent identification and resulting libraries were examined on an Agilent 2100 BioAnalyzer on a DNA 1000 chip and quantified by qPCR using NEBNext^®^ Library Quant Kit for Illumina (New England Biolabs). The libraries were sequenced using the NextSeq500 System from Illumina with the NextSeq 500/550 Mid Output v2 Sequencing kit (150 cycles, Illumina), thus guaranteeing 5 to 10 million paired-end reads per sample. Experiments were conducted in three repetitions, and the average results are shown.

### Bioinformatics

RNA sequencing data analysis was processed using a series of software scripts (Plocinski et al., 2019). The sequencing adapters were removed using Cutadapt software v.1.9.1 (Martin, 2011) and reads were qualitatively trimmed using the Sickle script with parameters maintaining a minimum quality of 30% and a minimum length of 20 bp. Trimmed reads were next mapped to the *M. smegmatis* genome (NC_008596.1, from NCBI; source: https://www.ncbi.nlm.nih.gov/nuccore/NC_008596) using Bowtie2 short sequence aligner (Langmead and Salzberg, 2012). Conversion and indexing were performed by the SAMtools software package (Li et al., 2009). Genes expression was considered different if the false discovery value (FDER) was <0.05, log2 fold change of >2.0. Gene ontology analysis was performed with an online tool ShinyGO v. 0.80 (http://bioinformatics.sdstate.edu/go) (Ge et al., 2020). Genome-wide *in silico* analysis of occurrences of the MnoR DNA-binding consensus was performed with FIMO of the meme-suite (Grant et al., 2011).

### Quantitative real-time PCR

For quantitative real-time PCR (qRT-PCR) experiments, RNA was extracted from wild-type *M. smegmatis* cells grown in the presence of 2 or 0.1% glucose using the TRIzol LS reagent (Invitrogen) as described previously (Pawelczyk et al., 2011); (Plocinska et al., 2022). For reverse transcription, a SuperScript III First-Strand Synthesis Super Mix (MP Biomedicals, Irvine, CA, USA) and random hexamers were used. qRT-PCR was performed using the PowerUp™ SYBR™ Green Master Mix (Applied Biosystems) and a QuantStudio™ 5 real-time PCR system (Applied Biosystems). Each reaction mixture (final volume 20 µl) was mixed on ice and contained 1 Maxima SYBR green qPCR master mix, 10 ng of cDNA, and 0.3 µM each primer (see **Supplementary Table S1** in the **Supplementary Material** for primer sequences). The reaction was carried out in several consecutive stages: initial activation (50°C for 2 min), initial denaturation (95°C for 2 min), 40-times repeated cycles: proper denaturation (95°C for 15 s), annealing (65°C for 15 s), extension (72°C for 1 nim). Data was acquired during the extension step. To verify the specificities and identities of the PCR products generated, a melting curve analysis was performed at the end of each PCR. The results were normalized with respect to the reference gene *msmeg_2758* (*mysA*) and then compared to the control strain. Each experiment was performed in triplicate, and the results are presented as the means and standard errors.

### Electrophoretic mobility shift assay

Interactions of His-MnoR with the Cyanine5 (Cy5)-labelled putative promoter regions of *mno* (perfect motif), *mno* motif with two mismatches (2MM), three mismatches (3MM) and four mismatches (4MM), were assessed using EMSA. First, 0, 0.5, 1, 2, 5 and 10 µM MnoR was incubated with 5 nM of labelled DNA in buffer containing 50 mM Tris-HCl, 100 mM NaCl, 10 mM MgCl_2_, 100 ug/ml BSA, 5% glycerol, 10 ng salmon sperm DNA. Additionally, similar reactions were prepared containing polyA at a concentration 100x higher than Cy5-labeled oligos. Samples were incubated for 30 min at 25°C and DNA-MnoR complexes were resolved on 5% nondenaturing polyacrylamide gels at 4°C. The protein-DNA complexes were visualized by Azure Imaging System 400. Oligonucleotide sequences used for EMSA are listed in **Supplementary Table S1**.

### Microscale thermophoresis

In order to determine whether the MnoR response regulator interact with the selected *mno* promoter sequences (perfect motif, motif with two (2MM), four (4MM) mismatches) microscale thermophoresis was performed. Binding affinities (Ki) were measured using the Monolith NT.115 from Nanotemper Technologies. The Cy5 labeled DNA oligonucleotides were synthesized by Sigma-Aldrich. Double stranded Cy5-DNA of *mno* perfect motif, *mno* 2MM and *mno* 4MM were diluted to reach an optimal fluorescence level between 200 to 2000 units of fluorescence. 60, 15, 120 nM of Cy5-*mno*, Cy5-*mno*2MM and Cy5-*mno*4MM respectively, were incubated for 5 minutes at room temperature with geometrically decreasing concentrations of MnoR protein (the protein was diluted from 27.5 µM down to 0.0008 µM). Reactions were performed in the PBS-T buffer and transferred to premium capillaries (Monolith). Measurements were performed at room temperature using 100% excitation power and 40% MST power. The fluorophores used in the procedure enable measurement of fluorescence, and on this basis the equilibrium dissociation constant (Kd) is determined for a given complex, determining the concentration at which half of the ligand (protein) is bound (Magnez et al., 2022). Data of three independent measurements were averaged and analyzed with the MO Affinity Analysis software. The sequences of the oligonucleotides used for microscale thermophoresis are listed in **Supplementary Table S1**. DNA used in microscale thermophoresis was sequence identical to the DNA used in EMSA.

### Statistical analysis

Statistical analyses in this study were carried out using GraphPad Software for Windows.

## Results

### The MnoSR 2CS is essential to utilize alcohols as the sole carbon source

The MnoSR two-component system was previously reported to be involved in the utilization of 1,3-propanediol by *M. smegmatis*. We asked whether the inactivation of MnoSR affects the global transcriptional profile of *M. smegmatis* growing in a carbon-free Sauton’s medium with 2% glucose as a carbon source and in a carbon-starvation condition (0.1% glucose). To answer this question, we used a two-step homologous recombination approach to construct *M. smegmatis* mutants with inactivated *mnoS*, *mnoR*, and both genes together. The genotypes of the resultant mutants (Δ*mnoS*, Δ*mnoR*, and Δ*mnoS/R*) were verified by PCR and Southern blot hybridization (**Supplementary Figure S1**). The transcripts of the *mnoS* and *mnoR* genes were also analyzed by RNA-seq.

The growth curves of the wild-type strain and mutants were compared in rich media (7H9/OADC) and carbon-free Sauton’s medium supplemented with 2% glucose, with no major differences (**Supplementary Figures S2A, B**).

**Figure 1:**
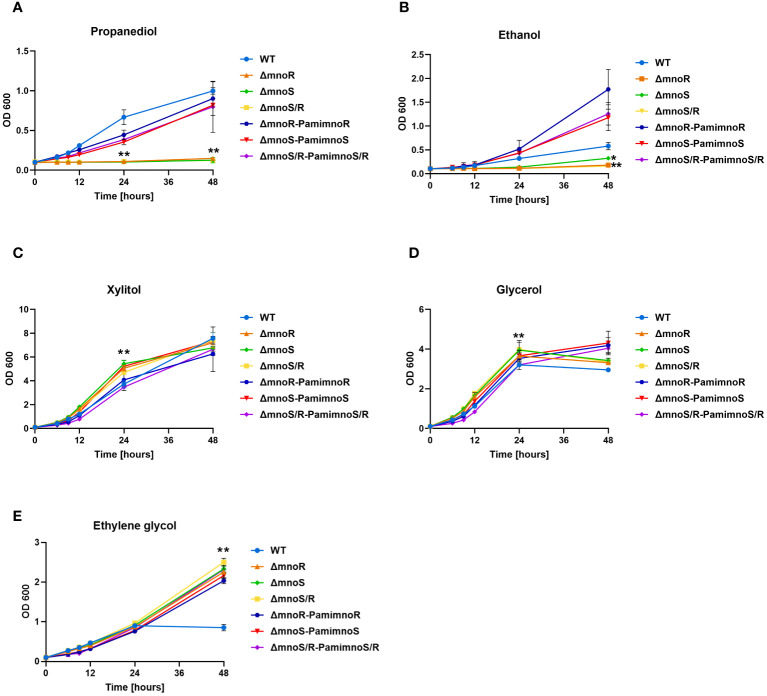
Phenotypic analyses of mutants defective in the synthesis of MnoSR two component system in *M. smegmatis*. Kinetics of growth of wild-type, mutants Δ*mnoS*, Δ*mnoR*, Δ*mnoS/R*, and complemented Δ*mnoS*-P*_ami_mnoS*, Δ*mnoR*-P*_ami_mnoR*, and Δ*mnoS*/*R*- P*_ami_mnoS*/*R M. smegmatis* strains cultured on carbon limiting Sauton’s medium containing 2% 1,3 propanediol **(A)**, 2% ethanol **(B)**, 2% xylitol **(C)**, 0.2% glycerol **(D)**, 2% ethylene glycol **(E)**. The growth of strains was evaluated by measuring the OD_600_ at the indicated time points. Means ± standard deviations are shown from three independent experiments. The statistical significance was determined using ordinary One-way ANOVA, Sidak’s multiple comparison test: for **(A)**, **(C)**, **(E)** (** *p* < 0.0001 for Δ*mnoS*, Δ*mnoR*, Δ*mnoS/R*); **(B)** (** *p* < 0.0001 for Δ*mnoR*, Δ*mnoS/R*), (* *p* = 0.0394 for Δ*mnoS*); **(D)** (** *p* < 0.0001 for Δ*mnoS*, Δ*mnoS/R*), (* *p* = 0.0234 for Δ*mnoR*).

To verify whether the inactivation of MnoSR affect the ability of the mutants to utilize alcohols, the wild-type strain, mutants (Δ*mnoS*, Δ*mnoR*, and Δ*mnoS*/*R*), and mutants complemented with intact genes (Δ*mnoS-P_ami_mnoS*, Δ*mnoR*-*P_ami_mnoR*, and Δ*mnoS/R*- *P_ami_mnoS/R*) were grown in carbon-free Sauton’s medium supplemented with 2% 1,3-propanediol (**Figure 1A**), 2% ethanol (**Figure 1B**), 2% xylitol (**Figure 1C**), 0.2% glycerol (**Figure 1D**) and 2% ethylene glycol (**Figure 1E**). We observed a significantly inhibited growth of mutant strains in comparison to wild-type and complemented strains in the presence of propanediol and ethanol. The kinetics of the growth evaluated in the presence of xylitol and glycerol in the media revealed a minor but significant increase in growth of mutant strains compared to wild-type. When ethylene glycol was the sole carbon source, we did not observe significant changes in the growth rate of the tested strains up to the 24-hour time point of the experiment. Interestingly, at the 48-hour time point, the growth of the wild-type cells measured by optical density (OD_600_) was inhibited, while the growth of the mutant strains continued to increase over time (**Figure 1**). To test whether the mutant strain would exhibit a longer lag phase of culture growth, we propagated wild-type, Δ*mnoS/R, and* Δ*mnoS/R-P_ami_mnoS/R* cells in carbon-free Sauton’s medium supplemented with 0.1% glucose. The cells were washed and the glucose was replaced with xylitol, glycerol, or ethylene glycol. We observed growth inhibition of the mutant strain compared to the wild-type up to the 12th hour of the experiment for each of the tested carbon sources. At the 24th hour, there was no difference in optical density among the tested strains for all three compounds. However, at the 48th hour, the growth of the mutant strain was significantly increased compared to the wild-type when xylitol and ethylene glycol were present in the medium. For glycerol, we did not observe differences at the 48th hour between the tested strains (**Supplementary Figure S3**).

### The MnoSR system is overexpressed during carbon starvation in *M. smegmatis*

**Figure 2:**
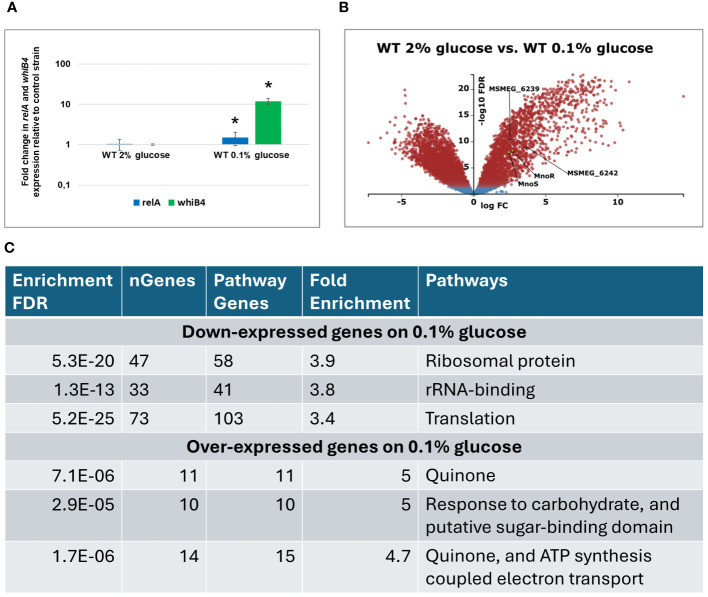
Transcriptomic profiling of *M. smegmatis* wild-type (WT) strain grown on carbon-rich (2% glucose) versus WT grown on carbon-limited (0.1% glucose) environment. Expression profile of the *relA* and *whiB1* genes in wild-type *M.smegmatis* strain grown in 7H9 minimal medium containing glucose. Total RNA was isolated and transcript levels were determined using qRT-PCR and SYBR green chemistry. The expression levels of *relA* and *whiB4* were normalized to the *mysA* housekeeping gene and compared to the control strain grown in carbon rich conditions. Mean values and standard deviations from three independent experiments are shown. The statistical significance was determined using Student’s *t*-test (P ≤ 0.05) **(A)**. Volcano plot analysis of transcriptomic profiling of *M. smegmatis* wild-type (WT) strain grown on carbon-rich (2% glucose) versus WT grown on carbon-limited (0.1% glucose) environment. The negative log of false discovery rate (FDR) (base 10) was plotted on the Y-axis, and the log of the fold change (FC) (base 2) was plotted on the X-axis. The red points represent transcripts that are differentially expressed (FC > 1 and FDR < 0.05). The blue points represent transcripts that are not differentially expressed **(B)**. The summary of gene ontology enrichment analysis performed with ShinyGO v.0.80 **(C)**.

As identified by whole genome analysis, the published palindromic target sequence for MnoR binding (Dubey and Jain, 2019) is present in a single copy in the chromosomal DNA of *M. smegmatis*, 137 bp upstream of *msmeg_6242* encoding alcohol dehydrogenase. To verify whether the inactivation of *mnoS/R* affects genes localized outside of the Mno operon (*msmeg_6242-6238*), we used a transcriptional profiling approach of wild-type *M. smegmatis* and Δ*mnoS/R* growing in minimal 7H9 media supplemented with 2% glucose as sole carbon source and under carbon-starvation condition – minimal 7H9 supplemented with 0.1% glucose. The original contributions presented in the study are publicly available. The RNA sequencing data can be found in the NCBI Bioproject database and are accessible at: https://www.ncbi.nlm.nih.gov/bioproject/PRJNA1121240. Actively growing cells from the exponential phase of growth were subjected to total RNA isolation and preparation of RNA sequencing libraries. Before starting the global RNA-Seq analysis, we examined whether the growth conditions used in the presence of 0.1% glucose led to the cells’ response to the carbon deficit in the medium. We selected two genes, *relA* and *whiB4*, previously described as up-regulated under carbon starvation conditions (Wu et al., 2016). qRT PCR analysis confirmed increased expression of *relA* (1.49 log 10 fold change (FC)) and *whiB4* (11.9 log10 FC) genes from *M. smegmatis* wild-type strain in carbon-limited environment (**Figure 2A**).

Transcriptomic analysis using KEGG-derived gene annotations (https://www.genome.jp/kegg/) and the Degust RNA-seq platform (https://degust.erc.monash.edu/) enabled a more precise identification of metabolic processes regulated by genes characterized by the greatest variation in expression between wild-type strain grown on glucose and during glucose starvation (0.1% glucose). The total of 3022 genes were differentially expressed in glucose-deprived cells compared to the same strain grown in the presence of 2% glucose (**Figure 2B**, **Supplementary Table S2**). Gene ontology analysis performed with ShinyGo 0.80 online analysis platform revealed that amongst 1561 down-expressed genes, the most enriched GO terms were related to ribosome and translation. Amongst 1462 upregulated genes, the same analysis revealed enrichment in GO terms related to oxidative phosphorylation and carbohydrate transport and metabolism (**Figure 2C**, **Supplementary Table S3**). The transcripts encoding *mnoS/R* and the *mno* operon were upregulated upon glucose starvation, presumably in preparation for utilization of alternative carbon sources that may be available in the growth environment as alternatives to glucose.

**Figure 3:**
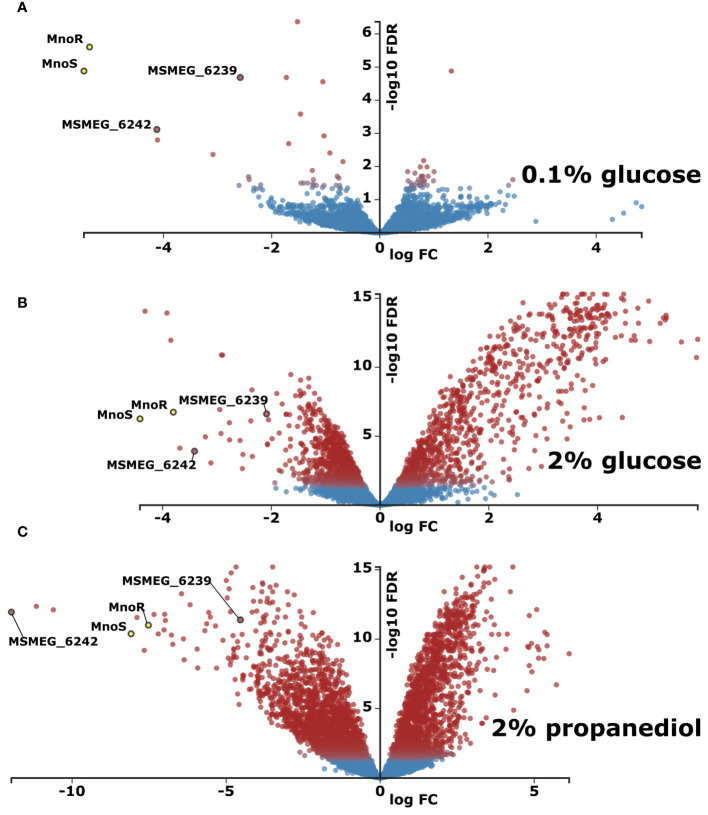
Transcriptomic profiling of *M. smegmatis* strain lacking *mnoS*/*R* genes in relation to wild type (WT) parental strain. Total RNA sequencing was performed from cells grown in carbon-limited environment of 0.1% glucose **(A)** carbon-rich environment of 2% glucose **(B)** or in 0.1% glucose with addition of 1,3 propanediol **(C)**. Presented as volcano plots were the negative log of false discovery rate (FDR) (base 10) was plotted on the Y-axis, and the log of the fold change (FC) (base 2) was plotted on the X-axis. The red points represent transcripts that are differentially expressed (Log2FC > |1.583| and FDR < 0.01). The blue points represent transcripts that are not differentially expressed. The log average expression level (base 10) correlates with the size of individual points.

The comparison of transcriptomics profiles of wild-type and *ΔmnoS/R* mutant strains grown in the carbon-limited conditions shown only 8 differentially expressed genes (Log2-fold change threshold set to ±1.58 with false discovery rate (FDR) <0.05) between the two strains, limited to the *mno* operon, with the addition of aldehyde dehydrogenase MSMEG_1543, propanediol dehydratase MSMEG_1546 and the 4-hydroxy-tetrahydrodipicolinate reductase MSMEG_0733, once again highlighting propanediol as one of the key metabolites that are affected by removal of *ΔmnoS/R* (**Supplementary Table S2**, **Figure 3A**). Thus, we decided to expand our initial model to follow transcriptional changes associated to *mnoS/R* removal also for cells grown in the presence of propanediol as a sole carbon source.

### Response of *ΔmnoS/R* mutant cells to carbon-rich environment

The multidimentional scaling plot (MDS) for RNA-Seq profiles of wild-type *M. smegmatis* and Δ*mnoS/R* mutant grown in 7H9 minimal media supplemented with 2%, 0.1% of glucose or 2% 1,3-propanediol in three independent biological experiments, showed that the replicates performed for each strain and for particular conditions clustered separately with high confidence. However, mutant lacking MnoSR system did not respond appropriately to the presence of alcohol in the culture media and shown similarities to the carbon-starved bacteria, clustering closer to the transcriptional profile of the wild-type and Δ*mnoS/R* strains starved in the absence of glucose (**Supplementary Figure S4**). Overall, transcriptional profiles of the Δ*mnoS/R* strain grown either on glucose or propanediol revealed numerous significant differences.

As expected, genes involved in regulation of methanol metabolism: the elements of two component system MnoSR (deleted) – response regulator *msmeg_6236* and sensor kinase msmeg_6238, along with the putative 1,3-propanediol dehydrogenase (*msmeg_6239*) and mno (*msmeg_6242*) were found down-regulated in all RNA-Seq analyses, whenever mutant strain was tested (**Supplementary Table S2**).

When grown in the presence of 2% glucose, Δ*mnoS/R* removal caused differential expression of 464 genes (421 upregulated and 43 downregulated, **Figure 3B**, **Supplementary Table S2**), many of which were involved in cellular processes related to carbohydrate metabolism, such as alpha-glucan catabolism, starch and sucrose metabolism, glucan metabolism as well as response to hypoxia (**Supplementary Table S3**). We observed a substantial down-regulation for the cluster of genes from *msmeg_2607* to *msmeg_2610* regulating the biosynthesis of cobalamin, cobalt transport protein CbiM and cobalt ABC transporter. The RNA-Seq analysis also revealed the lowered expression of *msmeg_2128* encoding malonyl CoA decarboxylase and the cluster of genes consisting of three genes *msmeg_ 2130, msmeg_2131, msmeg_2132* encoding acyl-CoA dehydrogenase, acyl-CoA synthase and acyl carrier protein Acp, respectively, thought to be involved in the biogenesis of the hydroxyphenyloxazoline-containing siderophore mycobactins. Additionally, in the top-three list of highest upregulated transcripts, *msmeg_1174* cadmium inducible protein cadi and two operon-encoded hypothetical genes: *msmeg_3937* and *msmeg_3938* were identified. Other important up-regulated genes grouped in operons included genes from the operon *msmeg_3265* to *msmeg_3273*, regulating carbohydrate metabolism. The largest cluster of upregulated genes ranged from *msmeg_1766* to *msmeg_1806*, with the exclusion of *msmeg_1776* and *msmeg_1795-1799*. However, most of the 33 significantly upregulated genes within the cluster present unknown functions. In addition to proteins of hypothetical function, the cluster contains genes encoding cytochrome P450 monooxygenase, SigF regulon elements: ChaB family protein (MSMEG_1802), RsbW (MSMEG_1803), the anti-sigma factor involved in partner switching system (PSS) of the SigF regulatory pathway (Oh et al., 2020) or RNA polymerase sigma-F factor. The inactivation of MnoSR significantly upregulated the expression of the arg operon consisting of seven genes (*msmeg_3770* to *msmeg_3776*) encoding proteins required for arginine biosynthesis (ArgG, ArgR, ArgF, ArgD, ArgB, ArgJ and ArgC), participating in nitrogen metabolism: nitrogen transfer in the urea Krebs cycle and in the synthesis of creatinine. The other large gene cluster consists of 30 highly upregulated ORFs from *msmeg_3926* to *msmeg_3955*. The cluster contains five universal stress proteins (MSMEG_3936, MSMEG_3939, MSMEG_3940, MSMEG_3945, MSMEG_3950), one of the three [NiFe] hydrogenases present in *M. smegmatis*, the Hyd3 (MSMEG_3931-MSMEG_3928), phosphoenolpyruvate synthase (PEPs) encoded by MSMEG_3934 converting pyruvate into phosphoenolpyruvate (essential step in gluconeogenesis when pyruvate or lactate are used as a carbon source), two component transcriptional regulatory protein DevR (MSMEG_3944), crucial for the survival of *Mycobacterium tuberculosis* during dormancy (Chauhan et al., 2011) and acyltransferase encoded by MSMEG_3948.

Even more differentially expressed genes were recorded when Δ*mnoS/R* mutant strain was grown on 2% 1,3-propanediol. In such a case, 1649 genes were differentially expressed, compared to the wild-type strain grown under the same conditions. Amongst 637 overexpressed genes were the ones related to oxidative phosphorylation and quinone metabolism, previously seen down expressed in the wild-type vs. wild-type carbon-starved comparison. On the contrary, transcripts encoding proteins related to ribosome biogenesis, RNA-binding and translation were enriched amongst 1012 genes found down expressed in the Δ*mnoS/R* strain (**Figure 3C**, **Supplementary Table S2**, **Supplementary Table S3**). This downregulation was previously recognized by us as a hallmark of carbon-starvation. Ribosome biogenesis-related downregulated gene clusters included *msmeg_1364-1365*, *msmeg_1398-1399*, *msmeg_1435-1446*, *msmeg_1465-1469*, *msmeg_1520-1525*, *msmeg_1556-1557*, *msmeg_2519-2520*, *msmeg_3791-3793, msmeg_4624-4625* as well as isolated ORFs *msmeg_1339, msmeg_1346, msmeg_1428, msmeg_2435, msmeg_2440, msmeg_2541, msmeg_2564, msmeg_4571, msmeg_4580, msmeg_4951, msmeg_5222, msmeg_5431, msmeg_5489, msmeg_6894, msmeg_6897 and msmeg_6946*.

### MnoRS weakly influences utilization of sugars as carbon sources and does not affect nitrogen metabolism

Next, we ask the question of whether the up- or downregulation of genes related to carbohydrate metabolism, observed in the MnoSR deficient strain, affected its ability to utilize various sugars as the sole source of carbon. Since genes related to carbohydrate metabolism (*msmeg_3265* to *msmeg_3273*) were upregulated in the mutant comparing to wild-type, when both grown on 2% glucose; and were down-regulated in the mutant comparing to wild-type, when both strains were starved on 0.1% glucose, we decided to investigate the potential impact of inactivation of MnoSR system on the ability of *M. smegmatis* cells to metabolize simple sugars and disaccharides as the sole carbon sources.

**Figure 4:**
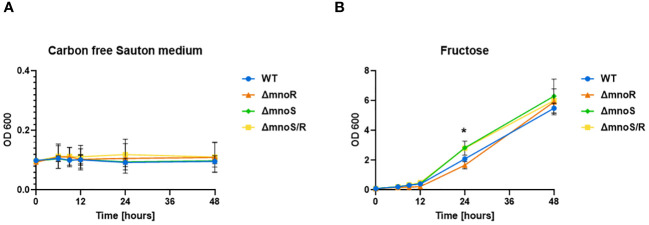
Kinetics of growth of mutant strains and wild type *M. smegmatis* propagated on carbon-limiting Sauton’s medium containing fructose. Wild-type, Δ*mnoS*, Δ*mnoR*, Δ*mnoS/R* were grown in the presence of fructose at 0.5% final concentration **(B)**. Carbon free Sauton’s medium was used as a negative control **(A)**. Growth of strains was determined by measuring the OD_600_ at indicated time points and was shown on the graphs as averages from three independent experiments ± standard deviation. The statistical significance was determined using Ordinary one-way ANOVA, Sidak’s multiple comparison test (** *p* = 0.0064 for Δ*mnoS*), (* *p* = 0.0240 for Δ*mnoS/R*).

For this purpose, we analyzed the growth rate of the tested strains on carbon-free minimal Sauton’s medium with the addition of fructose, mannose, lactose, maltose, ribose, xylose, and galactose at a final concentration of 0.5%. The growth of wild-type and mutant strains was efficient only in media supplemented with fructose (OD_600_ up to 6) and rather limited in the presence of other tested sugars (OD_600_ up to 1). No growth was recorded in the media devoid of carbon sources (**Figure 4A**). We observed a minor but statistically significant increase in the growth rate of the Δ*mnoS* and Δ*mnoS/R* mutants propagated in the medium containing fructose at 24 hour time point (*p*= 0.0064 for Δ*mnoS*, *p=* 0.0240 for Δ*mnoS/R*) (**Figure 4B**).

The kinetic of growth in the presence of other investigated sugars such as mannose, lactose, maltose, ribose, xylose, galactose was very much the same for wild-type and mutant strains (**Supplementary Figure S5**).

The inactivation of the tested genes did not affect the ability of *M. smegmatis* to metabolize the tested sugars, when utilized as a sole carbon source. Under glucose-rich conditions, in the mutant strain background, we observed high up-regulation of *msmeg_3934* encoding phosphoenolpyruvate synthase, the enzyme involved in pyruvate metabolism. We decided to check whether the Δ*mnoS/R* mutant strain could metabolize pyruvate as the only carbon source. We analyzed the kinetics of growth of mutants defective in the synthesis of MnoS, MnoR, and MnoSR in the presence of sodium pyruvate and sodium formate, as the sole carbon sources, at the final concentration of 0.5%. We observed no significant differences in the growth of mutants and wild-type strains in the presence of sodium pyruvate. Hence all tested strains were able to metabolize pyruvate as the only carbon source (**Supplementary Figure S6A**). On the other hand, neither strain was able to grow using sodium formate as the only carbon source (**Supplementary Figure S6B**).

In light of the observed overproduction of Arg proteins, we also attempted to check the kinetics of growth of *M. smegmatis* mutants lacking a functional MnoSR system on nitrogen-limiting Sauton’s medium, which contains various nitrogen sources. We tested the ability of the Δ*mnoS/R* strain to assimilate nitrogen from urea (pH=4.5), uric acid, histidine (pH 9.5), leucine (pH 9.5), proline (pH 9.5), ammonium sulphate, allantoin, hydantoin, methionine (pH 9.5), potassium L-glutamate, each at 10 mM final concentration and acetamide at 5 mM final concentration. The performed analysis revealed that inactivation of MnoSR does not affect the ability of *M. smegmatis* to utilize the tested nitrogen compounds. Weaker growth rates were recorded for all tested strains grown in the presence of leucine, hydantoin and methionine as the sole source of nitrogen (**Supplementary Figure S7**).

### DNA binding affinity of MnoR drops significantly with introduction of mutations into the MnoR-binding motif

As previously reported, applying electrophoretic mobility shift essay (EMSA), MnoR protein binds to the *mno* promoter region in a concentration-dependent manner (Dubey and Jain, 2019). In agreement to the published records, genome-wide *in silico* analysis with FIMO (meme-suite) revealed that the identified MnoR-consensus sequence is present in the genome of *M. smegmatis* in a single copy. We have also searched in *M. smegmatis* genome for MnoR-motif carrying up to four mismatches, compared to the published DNA-binding consensus. The analysis showed no sequence with a single mutation, 2, 14 and 97 sequences carrying two, three and four mismatches, respectively. Only 14 of these sequences fell within the intergenic regions, that could serve as putative promoter regions and modulate gene expression, however, strong correlation with gene expression and the presence of MnoR DNA-binding motif was only evident for *mno* operon.

To investigate whether MnoR could still target sequences containing mismatches, we performed EMSA analyzes with oligonucleotides reproducing the perfect DNA-binding consensus sequence and its variants with 2, 3 and 4 mutations (**Supplementary Figure S8A**). The full-length recombinant MnoR tagged with poly-histidine (6-His) at the N-terminus was expressed in *E.coli* and purified by affinity chromatography. As expected, MnoR bound efficiently to a Cy5-tagged consensus sequence that did not have any mutations. No interactions was observed between the tested protein and oligonucleotides with sequences containing two, three and four mismatches, when salmon sperm DNA was present in the reaction (**Supplementary Figure S8B**). Interestingly, when polyA was used instead of salmon sperm DNA, MnoR bound avidly to the all tested oligonucleotides containing mismatches (**Supplementary Figure S8C**).

Due to the ambiguous results obtained in EMSA assay we decided to determine the interaction strength of the MnoR protein with Cy5-labeled oligonucleotides possessing *mno* promoter region, sequences containing two or four mismatches by means of microscale thermophoresis (MST).

**Figure 5:**
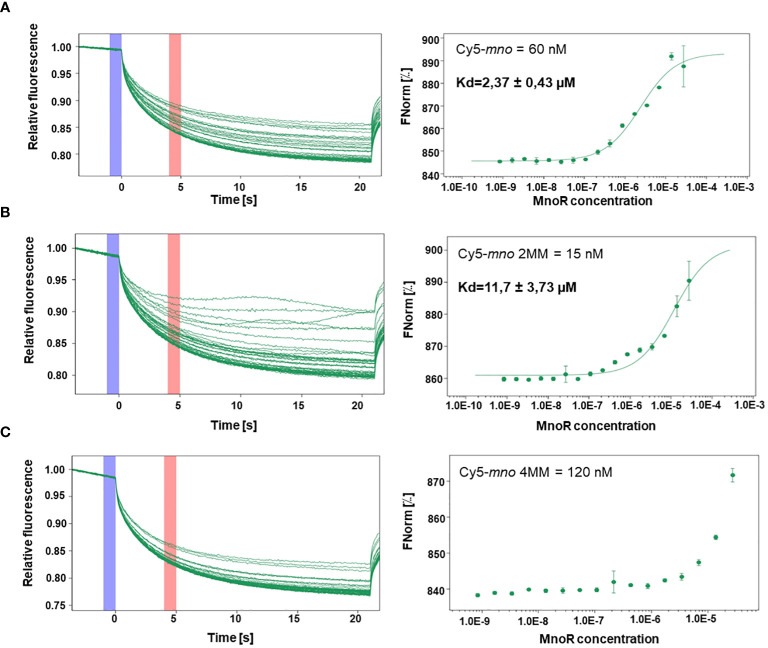
Interactions between MnoR response regulator and promoter region of *msmeg_6242* (*mno*) by microscale thermophoresis (MST). Perfect palindromic sequence or sequences carrying two (2MM) or four (4MM) mutations were analyzed by microscale thermophoresis on MonoLith NT.115. MST binding curves of MnoR with Cy5-*mno* perfect motif **(A)** Cy5-*mno* 2MM **(B)** and Cy5-*mno* 4MM **(C)**. The thermophoretic movement of a fluorescent molecule changes upon binding to a non-fluorescent ligand, resulting in different traces. Graphs are represented as Fnorm [‰] against ligand concentration. The error bars represent the standard deviation of each data point calculated from three independent thermophoresis measurements.

MST measurements showed binding of the MnoR protein to Cy5-*mno*, Cy5-*mno* 2MM. We observed that equilibrium dissociation constant (Kd) decreased with an increase in the number of mutations in the *mno* consensus sequence. Kd values were estimated to be 2.37 µM, 11.7 µM, respectively. MST results revealed the absence of affinity between MnoR and Cy5-*mno* 4MM DNA substrate (**Figure 5**).

## Discussion

The two-component signal transduction systems are crucial for bacterial adaptation to changing environmental conditions (Stock et al., 2000). Recent studies have shown that the MnoSR system regulates the expression of methanol dehydrogenase in *M. smegmatis* cells and affects the expression of the *msmeg_6239* gene, which encodes 1,3-propanediol dehydrogenase (Dubey and Jain, 2019). To further explore the role of the MnoRS system in mycobacteria, we investigated whether the MnoRS system solely regulates this specific operon or if it also influences the global cellular response to variable carbon sources. To this end, we constructed knockout mutant strains: Δ*mnoS*, Δ*mnoR*, and Δ*mnoS/R*, and assessed their growth in the presence of 1,3-propanediol and ethanol as sole carbon sources. As anticipated, the growth of these mutants was significantly reduced on media containing alcohols as carbon sources compared to the control strain. Our in silico analysis revealed that the consensus sequence for MnoR DNA-binding is present in only one copy in the *M. smegmatis* genome, with no sequences having a single mismatch. Additionally, our EMSA analysis and microscale thermophoresis confirmed that sequences with 2, 3, or 4 mismatches are weak substrates for MnoR protein binding *in vitro* without prior phosphorylation. This suggests that the MnoSR system specifically regulates a single operon in the *M. smegmatis* genome. To further validate our hypothesis, we conducted a comprehensive transcriptomic analysis of the Δ*mnoS/R* mutant under carbon starvation conditions and in the presence of glucose or propanediol as the sole carbon source. Performing transcriptomic analysis under specific environmental conditions enhances our understanding of adaptive processes during host infection or in response to therapy (Wang et al., 2009). This comparative transcriptomic analysis identified genes whose expression varied depending on carbon source availability and as a direct or indirect result of the MnoSR system inactivation. Although few genes showed differential expression between the mutant and wild-type cells under carbon starvation conditions with 0.1% glucose, we observed extensive changes in the transcriptomic profiles of these strains when cultured with 2% glucose or 2% 1,3-propanediol as the sole carbon source.

We initiated our global transcriptomic analysis by defining the carbon starvation response of *M. smegmatis*. For this purpose, we transferred exponentially grown bacteria to media containing 0.1% glucose, where they continued dividing for approximately 16 hours. We collected the cells once the culture density plateaued, treating it as an indicator of carbon exhaustion. We confirmed the induction of carbon starvation by monitoring the expression levels of *relA* and *whiB4* using quantitative real-time PCR. These genes have been identified as markers of resting cell formation (Wu et al., 2016) and RelA is a crucial enzyme in the stringent response, synthesizing the growth-regulating alarmone ppGpp to initiate cellular adaptation to nutrient starvation (Li et al., 2023). Although the cell density at collection was similar between the wild-type grown on 2% glucose and on 0.1% glucose, transcriptional analysis showed that nearly half of the transcriptome (3022 out of 6374 expressed genes) was differentially expressed under these two conditions. While the transcript for the primary RelA protein (MSMEG_2965) was moderately overexpressed (2.23-fold upregulation in RNA Sequencing) and did not exceed the 3-fold expression threshold set for our analysis, the secondary *relA* gene encoding functional (p)ppGpp synthetase (MSMEG_5849) (Murdeshwar and Chatterji, 2012) was upregulated more than 3-fold in our glucose starvation model. Notably, the WhiB4 transcriptional regulator was significantly overexpressed during carbon starvation, with over a 54-fold increase (5.77 Log2 Fold Change) in the RNA-Seq and a similar level in quantitative real-time PCR. *M. tuberculosis* WhiB4 has been reported to respond to oxidative stress and sense oxygen (O2) and nitric oxide (NO) levels via its 4Fe-4S cluster (Chawla et al., 2012). Reports suggest that WhiB4 could be regulated by SigF, an alternative sigma factor involved in complex regulatory mechanisms in response to various stresses in *M. tuberculosis* (Oh et al., 2020). In our analysis, SigF (MSMEG_1804) was overexpressed more than 4-fold, and the SigF anti-sigma factor RsbW (MSMEG_1803) was upregulated by more than 28-fold. Another potential target for SigF-dependent regulation, MSMEG_5542 (an HTH3 family transcription factor), was also among the most strongly upregulated genes, with its expression during carbon starvation reaching more than 97-fold of the level recorded under carbon-rich conditions. Other candidates for SigF-dependent regulation, like MSMEG_1919, remained relatively unchanged, or were downregulated (MSMEG_5731, MSMEG_6508) (Singh et al., 2015). Gene ontology analysis identified ribosome biogenesis and translation as the processes most negatively affected in our carbon starvation model.

Recent research has illuminated the mechanisms through which RelA interacts with translating ribosomes and detects deacylated-tRNA at the A-site of the stalled ribosome complex in a nutrient-starved cell (Li et al., 2009). A significant number of genes encoding structural constituents of the ribosome, 53 out of 66, were markedly down expressed in carbon-starved *M. smegmatis*. To the best of our knowledge, no studies have detailed the precise mechanism by which a large group of genes responsible for coding ribosomal proteins can be downregulated simultaneously in mycobacterial models. Extensive research on *E. coli* has shown that RNA structures within transcripts encoding ribosomal proteins interact with specific ribosomal proteins. This interaction facilitates autogenous regulation, allowing for the adjustment of expression within extensive multi-gene operons and thereby coordinating the biosynthesis of ribosomal proteins across various genomic loci (Fu et al., 2013).

The differential gene expression between carbon starved wild-type strain and the mutant strain grown under the same conditions, revealed that only 8 genes were changed, with all of them being down expressed in the mutant strain including *mno* operon, MnoSR system itself (deleted and thus recognized as downregulated) and three more genes being aldehyde dehydrogenase MSMEG_1543, propanediol dehydratase MSMEG_1546 and the 4-hydroxy-tetrahydrodipicolinate reductase MSMEG_0733. The *in silico* analysis with FIMO failed to discover MnoR DNA-binding sites in the promoter regions for these genes with 4 or less mutations to the original motif sequence, which we considered as the maximum number of mismatches allowed to secure DNA-binding based on our EMSA and Microscale Thermophoresis assays.

Global analysis of the transcriptomes of the wild-type and the mutant cells grown in the presence of carbon sources showed broad transcriptional differences between the tested strains. When glucose was supplemented as a sole source of carbon at 2% concentration, significant expression changes were recorded for the mutant strain showing 464 differentially expressed genes. Most of these changes were upregulations, accounting for 421 changes, with 43 remaining differences being down-expressions. Gene Ontology analysis revealed response to carbohydrate was strongly enriched in the group of overexpressed genes, and cobalt ion transport as well as siderophore biogenesis were enriched pathways in the down expressed gene set.

Although we did not observe changes in the growth kinetics of the wild-type strain and the mutant on glucose, we could not rule out that sugar metabolism is affected in the absence of MnoRS. Therefore, we assessed the ability of the tested strains to grow on a medium with the addition of other simple sugars as the sole carbon sources, observing a temporary, yet significant, increase in the growth rate of the mutant strains grown on fructose. Fructose was the only other sugar, apart from glucose, efficiently utilized by *M. smegmatis*, allowing the bacterial cells to reach higher densities than the other sugars tested in our work. Our data support the conclusions drawn by Baloni’s team that transcriptomic and phenotypic analyses of *M. smegmatis* allow mapping of the existence of various, often alternative, pathways for utilizing different compounds, which can be activated under specific conditions (Baloni et al., 2014). In the presence of 2% glucose, a significant overexpression, reaching 38 fold, of the *msmeg_3934*, encoding phosphoenolpyruvate synthase was observed in the mutant strain. This enzyme is involved in the transformation of pyruvate into phosphoenolpyruvate, which is an essential step in the process of gluconeogenesis, where pyruvate or lactate are the carbon sources. McCormick and Jakeman showed that PEPs are necessary for the growth of *E. coli* on three-carbon sources such as pyruvate (McCormick and Jakeman, 2015). Research conducted by Zhang confirmed the presence of phosphoenolpyruvate synthase in *M. smegmatis* cells and the involvement of its product in gluconeogenesis (Zhang and Anderson, 2013). Analysis of growth kinetics in the presence of sodium pyruvate showed that both the mutant and the wild-type strains are able to metabolize it. The ability to use pyruvate as a carbon source has also been described for *M. tuberculosis*. Analyzes of the pyruvate transformation pathway conducted by Verma’s team indicate the existence of alternative pyruvate degradation pathways in actinobacterial cells (Verma et al., 2013). The lack of differences in the growth kinetics of MnoSR mutants compared to the wild-type strain in the presence of pyruvate most likely indicates the existence of alternative ways of metabolizing this carbon source in *M. smegmatis* cells and the complexed metabolic networks that make up the glycolysis process. A closer inspection of the genomic region encoding phosphoenolpyruvate revealed that the entire region from *msmeg_3926 to msmeg_3955* is overexpressed in the mutant strain grown in the presence of 2% glucose, including DevR and DevS two-component system, controlling the expression of numerous genes in this region, and being the major regulator of hypoxic responses in mycobacteria (Trauner et al., 2012).

Another important observation was that removal of MnoSR in the glucose-rich environment led to a significant reduction of expression of the *msmeg_2607*, *msmeg_2608* and *msmeg_2609* encoding cobalamin biosynthesis protein, cobalt transport protein and cobalt ABC transporter, respectively. Cobalamin (vitamin B12) is considered an essential cofactor and is synthesized only by certain bacteria and archaeons (Watanabe and Bito, 2018), including non-tuberculous mycobacteria strains (Minias et al., 2021). Cobalamin biosynthesis *de novo* is a complex multistep process and requires at least 25 unique enzymes in 20 energy-consuming enzymatic reactions, leading to the rare bonding of carbon to the cobalt atom (Martens et al., 2002). *M. tuberculosis* carries approximately 30 genes predicted to be involved in cobalamin biosynthesis. Cobalamin affects the metabolism of *M. tuberculosis* through two mechanisms: acting as a cofactor for three enzymes methionine synthase (MetH), methylmalonyl-CoA mutase (MCM) and ribonucleotide reductase NrdZ (Savvi et al., 2008); (Warner et al., 2007)), and also by regulating gene expression by attaching to riboswitches in mRNA. Recently, *de novo* cobalamin biosynthesis was confirmed in *M. smegmatis* during aerobic growth *in vitro* (Kipkorir et al., 2021).

The transcriptomic analysis also revealed the influence of the MnoSR system on arginine metabolism in carbon-rich conditions. Arginine, a source of carbon and nitrogen, is crucial for the survival of *M. tuberculosis* cells in the host (Cui et al., 2022). The arginine biosynthetic pathway is regulated by eight enzymes, including the enzyme N-acetyl-gamma-glutamyl phosphate reductase encoded by *argC* (*rv1652*, *msmeg_3776*), a gene necessary for the growth of *M. tuberculosis in vitro* (Gupta et al., 2021). In our studies, we did not observe the effect of inactivation of the MnoSR system on the growth of strains in the presence of various nitrogen compounds such as urea, uric acid, histidine, leucine, proline, ammonium sulphate, allantoin, hydantoin, methionine, potassium L-glutamate, acetamide.

Our study confirmed the role of the MnoSR two-component system in managing the metabolic adaptability of *Mycobacterium smegmatis* to fluctuating environmental carbon sources. The MnoSR system is crucial for the utilization of specific alcohols, like 1,3-propanediol, as sole carbon sources, illustrating its specific regulatory function in methylotrophic metabolism. Additionally, the global transcriptomic analysis under conditions of carbon limitation, glucose or propanediol, revealed extensive changes in gene expression, underscoring the system’s influence beyond methylotrophy, affecting various metabolic processes crucial for survival under nutrient-limited conditions. These findings emphasize the complexity of bacterial adaptation mechanisms and could inform strategies to target metabolic pathways in pathogenic mycobacteria.

## Data availability statement

The RNA sequencing data can be found in the NCBI Bioproject database and are accessible at: https://www.ncbi.nlm.nih.gov/bioproject/PRJNA1121240.

## Author contributions

RP: Conceptualization, Funding acquisition, Investigation, Visualization, Writing – original draft. KS: Conceptualization, Investigation, Visualization, Writing – original draft. MK-M: Conceptualization, Investigation, Visualization, Writing – original draft. PP: Conceptualization, Investigation, Visualization, Writing – original draft. MK: Investigation, Writing – original draft. AŻ: Conceptualization, Funding acquisition, Writing – original draft. MS: Investigation, Writing – original draft. JD: Conceptualization, Supervision, Writing – review & editing.
