# Supplementary material for: MnoSR removal in Mycobacterium smegmatis triggers broad transcriptional response to 1,3-propanediol and glucose as sole carbon sources
Source: Front Cell Infect Microbiol. 2024 Jul 24;14:1427829. doi: 10.3389/fcimb.2024.1427829 (PMC11303327; doi:10.3389/fcimb.2024.1427829)
Supplement: Supplementary file 1 [file DataSheet_1.pdf]

# Supplementary materials

**Title: MnoSR removal in *Mycobacterium smegmatis* triggers broad transcriptional response to 1,3-propanediol and glucose as sole carbon sources**


Renata Płocińska#[1], Katarzyna Struś#[1], Małgorzata Korycka-Machała[1], Przemysław Płociński[2], Magdalena Kuzioła[1,3], Anna Żaczek[4], Marcin Słomka[5], Jarosław Dziadek[1]*

[1] Institute of Medical Biology of the Polish Academy of Sciences, Lodowa 106, 93-232 Łódź, Poland

[2] Department of Immunology and Infectious Biology, Faculty of Biology and Environmental Protection, University of Łódz, Banacha 12/16, 90-237 Łódź, Poland

[3] BioMedChem Doctoral School of the University of Łódź and Łódź Institutes of the Polish Academy of Sciences, Matejki 21/23, 90-237 Łódź, Poland

[4] Department of Microbiology, College of Medical Sciences, University of Rzeszów, Rejtana 16c, 35-959 Rzeszów, Poland

[5]Biobank Lab, Department of Molecular Biophysics, Faculty of Biology and Environmental Protection, University of Łódź, Pomorska 139, 90-235 Łódź, Poland

# These authors has contributed equally to this work

* Correspondence should be addressed to jdziadek@cbm.pan.pl



**Keywords:** histidine sensor kinase MnoS, response regulator MnoR, Mycobacterium smegmatis, methylotrophic metabolism, RNA-seq

**Table of contents:**

**1. Table S1** List of strains, plasmids and primers used in this study.

**2. Table S2 Global transcription analyzes using RNA-seq** (separate excel file associated with this manuscript)**. (A)** Genes differentially expressed between wild-type strain grown exponentially in 7H9 media supplemented with 2 % glucose and wild-type grown in 7H9 media supplemented with 0.1 % glucose. **(B)** Genes differentially expressed between Δ*mnoS*/*R* and wild-type strains grown exponentially in 7H9 media supplemented with 0.1 % glucose - carbon-limited environment. **(C)** Genes differentially expressed between Δ*mnoS/R* and wild-type strains grown exponentially in 7H9 media supplemented with 0.1 % glucose and 2 % 1,3 propanediol as sole carbon source. **(D)** Genes differentially expressed between Δ*mnoS*/*R* and wild-type strains grown exponentially in 7H9 media supplemented with 2 % glucose - carbon-rich environment.

**3. Table S3 Gene oncology enrichment** (separate excel file associated with this manuscript).

**4. Supplementary Figures** with corresponding legends.

**Table S1** List of plasmids, strains and oligonucleotides used.

| **A. Oligonucleotides used in this study** | | |
|---|---|---|
| Name | Sequence (5’>3’) | Application |
| **Primers used for cloning** | | Amplification region |
| Ms6238GR1bScaI-s | cagtactCGTTGACAAGGCCACCATGC | *Ms-mnoS* upstream region (47 bp) including 5’ of *mnoS* gene (1152 bp) |
| Ms6238GR2HindIII-r | caagcttCATCGAGGATGAGGTGACGC | |
| Ms6238GR3bHindIII-s | caagcttGGCACGCAGGATCTGGTCGA | *Ms-mnoS* downstream region (546 bp) including 3’ of *mnoS* gene (1152 bp) |
| Ms6238GR4bBamHI-r | cggatcCGGTGGTCGCGGAGATGAAC | |
| Ms6236GR1b-ScaI-s | cagtactACGGCCCACCGAAACTCGTG | *Ms-mnoR* upstream region (89 bp) including 5’ of *mnoR* gene (651 bp) |
| Ms6236GR2b-HindIII-r | caagctTCACGTTCCAGCAGCGA | |
| Ms6236GR3HindIII-s | caagcttTGACAGTGCGTTCGACTCCCG | *Ms-mnoR* downstream region (112 bp) including 3’ of *mnoR* gene (651 bp) |
| Ms6236GR4BamHI-r | cggatccGCGCACCGCGACTAGTTCGT | |
| Ms6238BglII-s | cagatctGTGGCCGAAGCGGCCCGCAC | Confirmation of the genotype of the Δ*mnoS SCO/DCO mutant* |
| Ms6238XbaI-r | ctctagaTTATCGTCTGTTCCCTTCGGTC CTGG | |
| Ms6236BamHI-s | cggatccATGACCGTCACGACGCGCGAG | Confirmation of the genotype of the Δ*mnoR SCO/DCO mutant* |
| Ms6236XbaI-r | ctctagaTTAGATCAACCCGCGCTTGCTC G | |
| Ms6236BamHI-s | cggatccATGACCGTCACGACGCGCGAG | *Msmeg-mnoR* gene to clone into pMV306- under $P_{ami}$ promoter |
| Ms6236XbaI-r | ctctagaTTAGATCAACCCGCGCTTGCTC G | |
| Ms6238BglII-s | cagatctGTGGCCGAAGCGGCCCGCAC | *Msmeg-mnoS* gene to clone into pMV306- under $P_{ami}$ promoter |
| Ms6238XbaI-r | ctctagaTTATCGTCTGTTCCCTTCGGTC CTGG | |

| | | |
|---|---|---|
| MsmnoR-s | cggatccgatgaccgtcacgacgcgcgagatc | *Msmeg-mnoR* gene to clone into pHIS expression vector |
| MsmnoR-r | caagcttctagatcaacccgcgcttgctcg | |
| **Primers used for qRT-PCR** | | |
| Ms6236RTPCR-s | AAGTCCGATCCGGCCGAGAGC | Gene expression analysis *mnoR* |
| Ms6236RTPCR-r | CGCTGCTGGAACGTGAGGACG | |
| Ms6238RTPCR-s | ACCGGAACTGCGCCAGGTGC | Gene expression analysis *mnoS* |
| Ms6238RTPCR-r | CTCATCCTCGATGCCGACGGG | |
| MsSigARTPCR-s | CCAAGGGCTACAAGTTCTCG | Gene expression analysis *sigA* |
| MsSigARTPCR-r | TGGATCTCCAGCACCTTCTC | |
| MswhiB4RTPCR-s | AGCACCGGGACTCCGAAGCG | Gene expression analysis *msmeg_1831* |
| MswhiB4RTPCR-r | CGATTGTCGAGCGCGTCCG | |
| MsrelARTPCR-s | TGTCCAGCTTGGTCACACCGTCG | Gene expression analysis *msmeg_5571* |
| MsrelARTPCR-r | AGCTCGGCATGGACACCACCAC | |
| **Primers used for EMSA** | | |
| MnoR-motif-perfect-s | Cy5-gcctgagcgatctggggctctgagcaccctcgcccaccggtgagccgccggtgggcggccaccctgcccgaccggacagttctgccg | |
| MnoR-motif-perfect-r | cggcagaactgtccggtcgggcagggtggccgcccaccggcggctcaccggtgggcgagggtgctcagagccccagatcgctcaggc | |
| MnoR-motif-2MM-s | Cy5-acctggcggatctgagcccggcaggcgatccgcacaccggtaccgtgctggtgggcgcgagccccgagttgctggtcgcccgcgacg | |
| MnoR-motif-2MM-r | cgtcgcgggcgaccagcaactcggggctcgcgcccaccaagcacggtaccggtgtgcggatcgcctgccgggctcagatccgccaggt | |

| | | |
|---|---|---|
| MnoR-motif-3MM-s | Cy5-gctggaggaggcgttcaccgctgactggctcgcccagcactcctaagccggtgggcgccgtcacccgcggcaccaccggttacaacc | |
| MnoR-motif-3MM-r | ggttgtaaccggtggtgccgcgggtgacggcgcccaccggcttaggagtgctgggcgagccagtcagcggtgaacgcctcctccagc | |
| MnoR-motif-4MM-s | Cy5-cacgctgatcacgtccgacgtcgacgagcgcgaccagccgttcatcaccggtgagcgcaccaaggaaggcttcttccgcgtgaagaa | |
| MnoR-motif-4MM-r | ttcttcacgcggaagaagccttccttggtgcgctcaccggtgatgaacggctggtcgcgctcgtcgacgtcggacgtgatcagcgtg | |
| **B. Strains used in this study** | | |
| **Name** | **Description** | **Reference** |
| Top10F’ | *Escherichia coli* strain | Invitrogen |
| Mc$^2$155 | *M. smegmatis* wild type | Laboratory stock |
| BL21 (DE3) | *E. coli* strain | Novagen |
| Δ*mnoS* | *M. smegmatis mnoS* mutant | This study |
| Δ*mnoR* | *M. smegmatis mnoR* mutant | This study |
| Δ*mnoS/R* | *M. smegmatis mnoS/R* mutant | This study |
| Δ*mnoS*-$P_{ami}$*mnoS* | *M. smegmatis* Δ*mnoS* mutant, carrying *mnoS* with internal deletion, complemented with *mnoS* gene under $P_{ami}$ promoter in *attB* site | This study |
| Δ*mnoR*-$P_{ami}$*mnoR* | *M. smegmatis* Δ*mnoR* mutant, carrying *mnoR* with internal deletion, complemented with *mnoR* gene under $P_{ami}$ promoter in *attB* site | This study |
| Δ*mnoS/R*-$P_{am2}$*mnoS/R* | *M. smegmatis* Δ*mnoS/R* mutant, carrying *mnoS/R* with internal deletion, complemented with *mnoS/R* gene under $P_{ami}$ promoter in *attB* site | This study |

| | | |
|---|---|---|
| | | |
| **C. Plasmids used in this study** | | |
| pJET 1.2/blunt | Blunt cloning vector, $Amp^R$, for subcloning of gene fragments | Thermo Scientific |
| p2NIL | Suicidal gene delivery vector, nonreplicating in mycobacteria, $Kan^R$ | (Parish & Stoker, 2000) |
| pGoal17 | Source of PacI cassette, $Amp^R$ | (Parish & Stoker, 2000) |
| pMV306K | Mycobacterial integrating vector, $Kan^R$ | Med-Immune Inc |
| pJAM2 | Mycobacterial integrating vector, $Kan^R$ | (Triccas et al., 1998) |
| pHIS | *E. coli* expression vector enabling the purification of HIS-tagged protein, $Amp^R$ | (Sheffield et al., 1999) |
| pKS23 | pHIS carrying *mnoR* | This study |
| pKS10 | p2NIL carrying Δ*mnoS* with flanking regions and PacI cassette from pGOAL17 | This study |
| pKS11 | p2NIL carrying Δ*mnoR* with flanking regions and PacI cassette from pGOAL17 | This study |
| pKS12 | p2NIL carrying Δ*mnoSR* with flanking regions and PacI cassette from pGOAL17 | This study |
| pKS19 | Mycobacterial integrating vector carrying *M. smegmatis mnoR* under $P_{ami}$ promoter | This study |
| pKS20 | Mycobacterial integrating vector carrying *M. smegmatis mnoS* under $P_{ami}$ promoter | This study |
| pKS22 | Mycobacterial integrating vector carrying *M. smegmatis mnoSR* under $P_{ami}$ promoter | This study |

**Figure S1.**

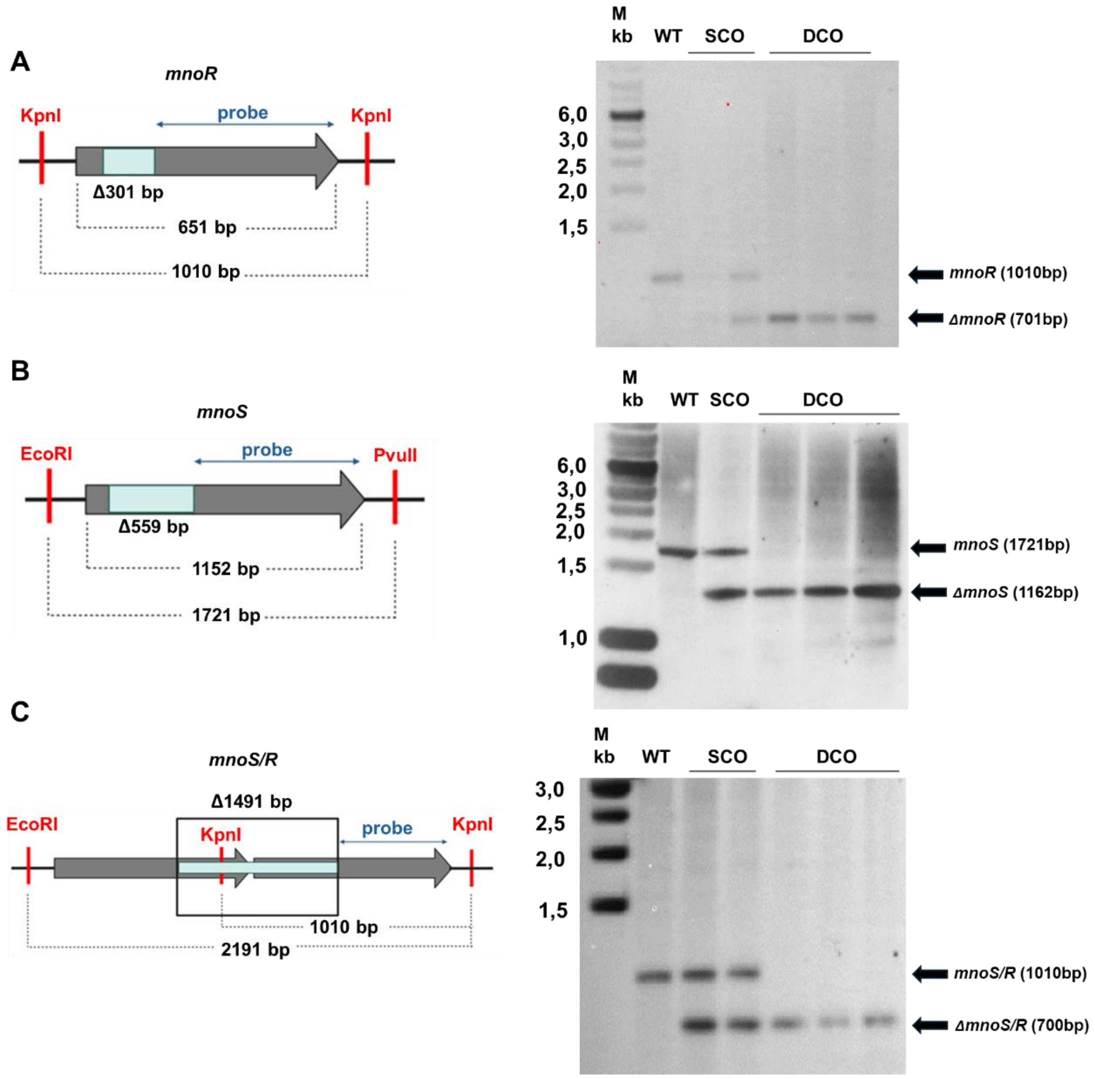


**Figure S1.** Genotype analysis of mutants by Southern blot hybridization. Genomic organization of *mnoR* gene with restriction sites used for digestion of chromosomal DNA and Southern blot hybridization of chromosomal DNA isolated from wild-type *M. smegmatis* (WT), single cross-over (SCO) and double cross over (DCO) **(A)**. Genomic organization of *mnoS* gene with restriction sites used for digestion of chromosomal DNA and Southern blotting analysis of chromosomal DNA isolated from wild-type *M. smegmatis* (WT), SCO and DCO **(B)**. Genomic organization of *mnoS/R* genes with restriction sites used for digestion of chromosomal DNA and Southern blotting analysis of chromosomal DNA isolated from wild-type *M. smegmatis* (WT), SCO and DCO **(C)**. M - 1kb ladder.

**Figure S2.**

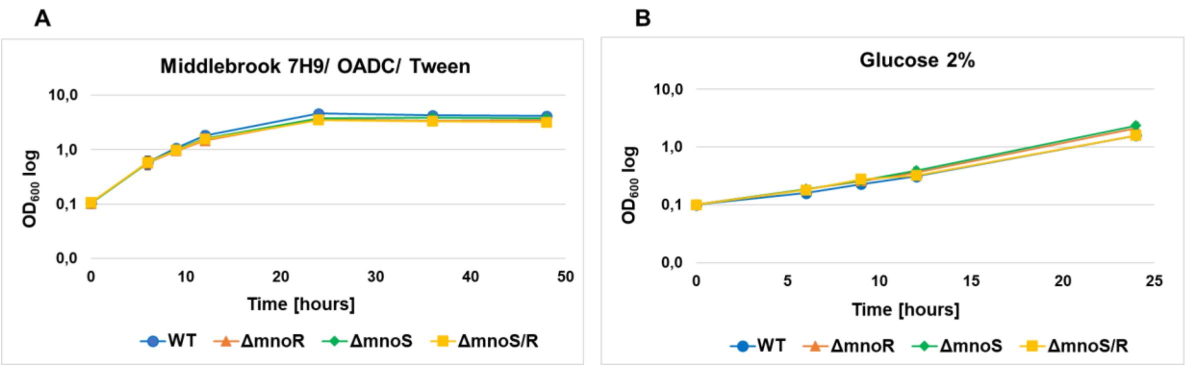


**Figure S2. Phenotypic analyses of mutants defective in the synthesis of MnoSR two component system in *M. smegmatis*.** Kinetics of growth of Δ*mnoS*, Δ*mnoR*, Δ*mnoS/R* and wild-type *M. smegmatis* strains cultured in rich media, Middlebrook 7H9 supplemented with OADC **(A)** and carbon limiting Sauton's medium containing 2% glucose **(B)**. The growth of strains was evaluated by measuring the $OD_{600}$ at the indicated time points. The displayed values are the average ± standard deviation from three independent experiments.

## Figure S3

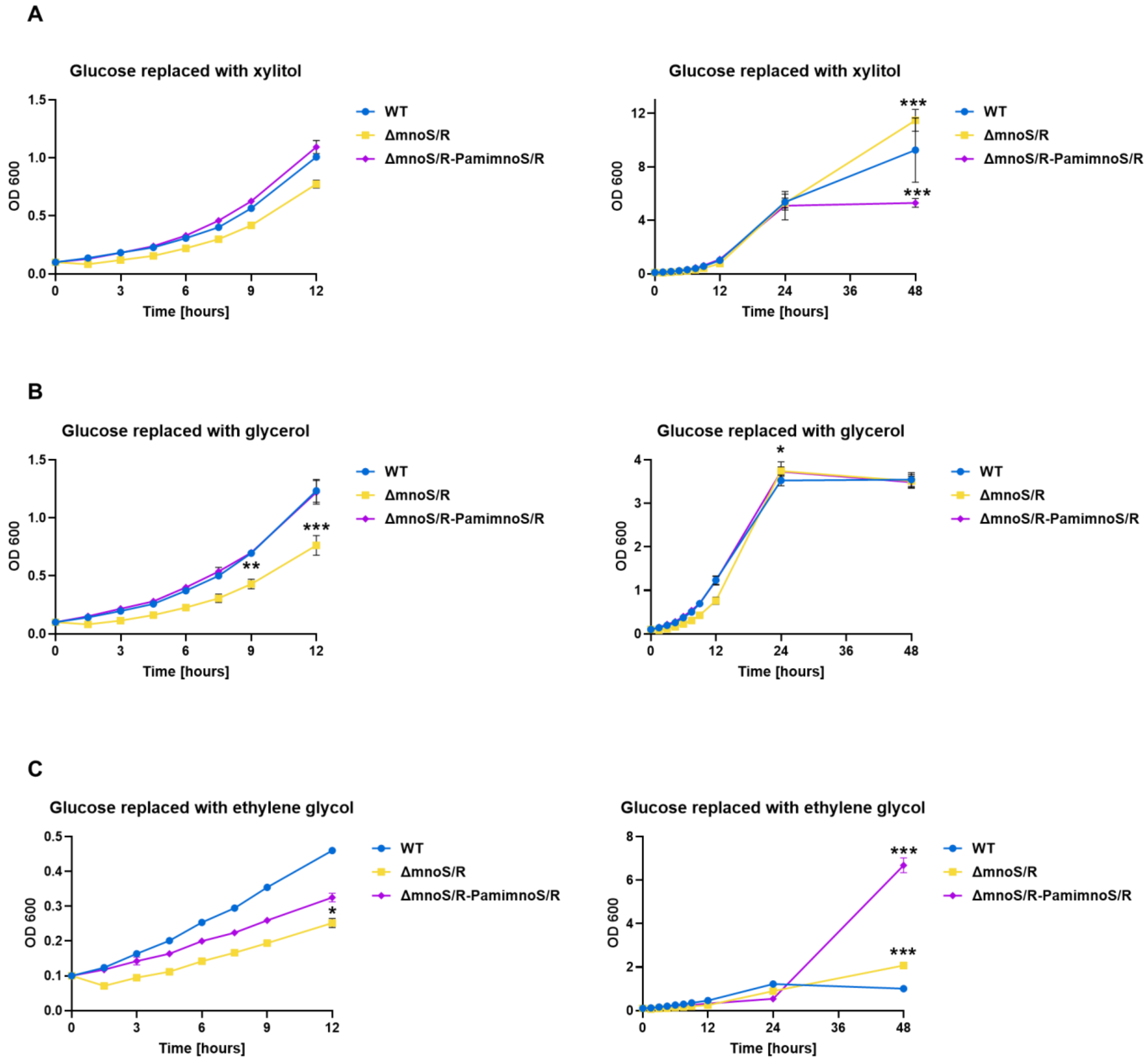


**Figure S3**. Kinetics of growth for mutant strains and wild-type *M. smegmatis* propagated in carbon-limiting Sauton's medium, where glucose was replaced with xylitol (A), glycerol (B), and ethylene glycol (C). To monitor optical density measurements during the lag phase of growth, the data are presented in two graphs: one up to 12 hours and another up to 48 hours. The statistical significance was determined using Ordinary one-way ANOVA, Sidak's multiple comparison test: *** $p < 0.0001$, (B) * $p = 0.0179$ for Δ*mnoS/R*, ** $p = 0.015$ for Δ*mnoS/R*, (C) * $p = 0.0136$ for Δ*mnoS/R*.

**Figure S4.**

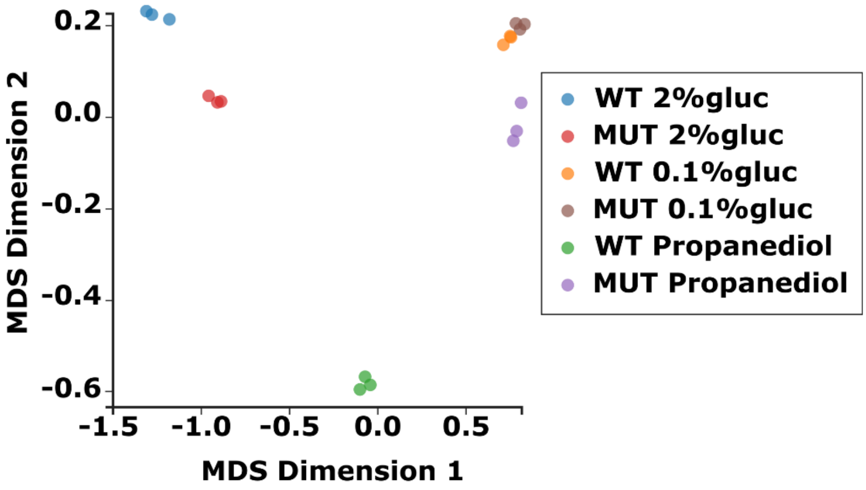


**Figure S4. Multidimensional Scaling (MDS) analysis of the repeats of wild-type (WT) and Δ*mnoS/R* (MUT) strains used in RNA-seq analyzes**. Each repeat of the individual strain is marked by blue, red, orange, brown, green and purple dots. The results were obtained using Degust online data analysis platform.

**Figure S5.**

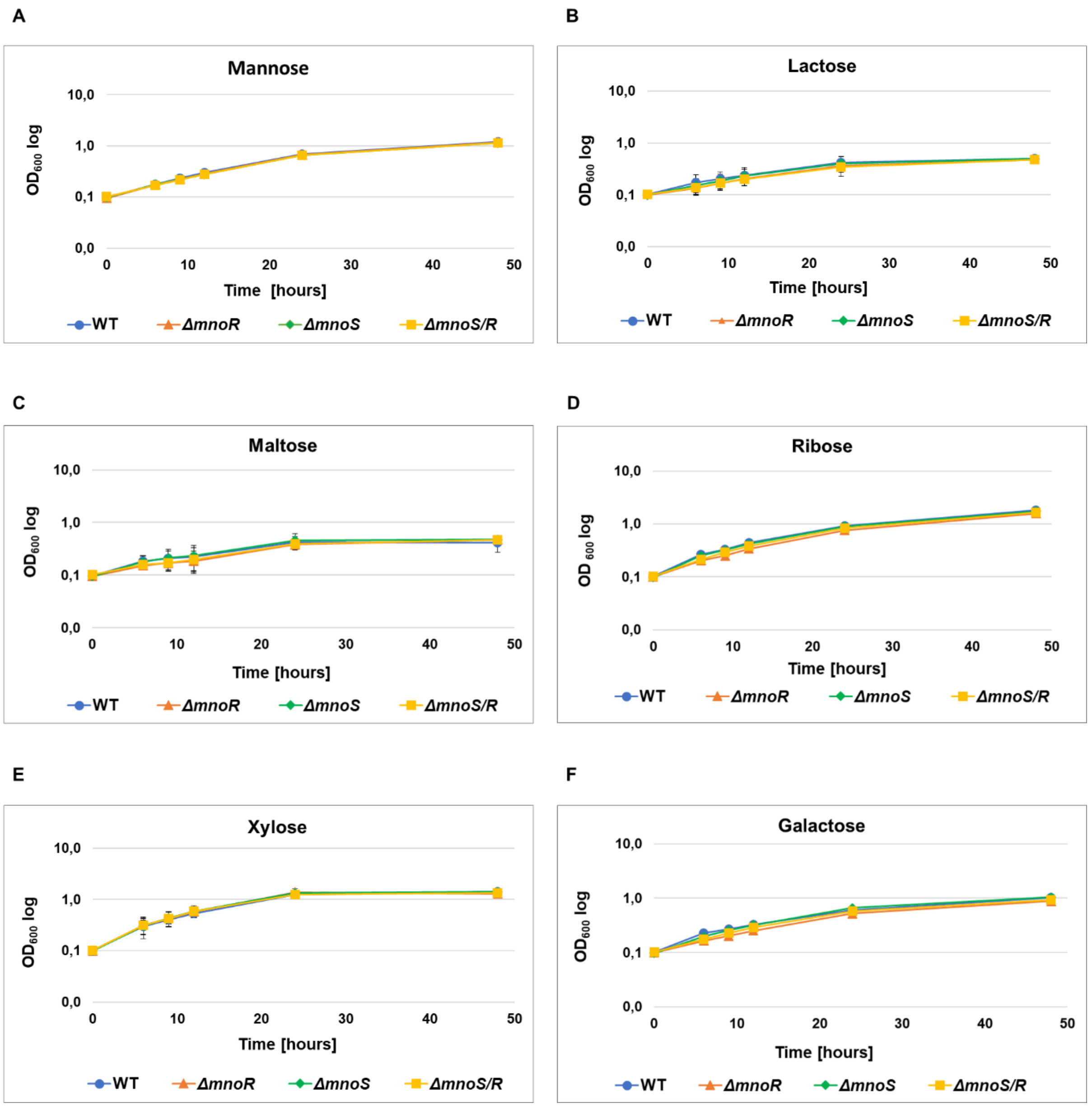


**Figure S5. Kinetics of growth of mutant strains and wild-type *M. smegmatis* propagated on carbon-limiting Sauton's medium containing various carbon sources.** Wild-type, Δ*mnoS*, Δ*mnoR*, Δ*mnoS/R* were grown in the presence of the mannose (A), lactose (B), maltose (C), ribose (D), xylose (E) and galactose (F) at final concentration 0.5%. Growth of strains was determined by measuring the $OD_{600}$ at indicated time points and was shown on the graphs as averages from three independent experiments ± standard deviation.

**Figure S6**.

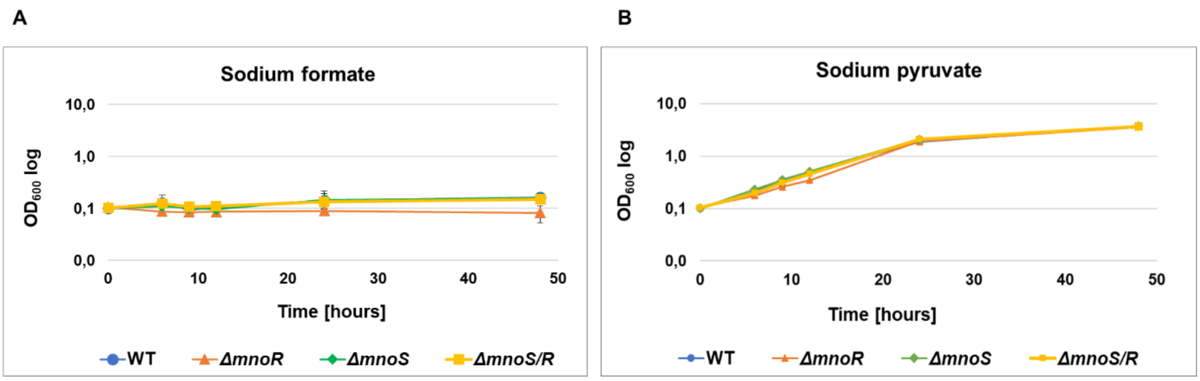


**Figure S6. Kinetics of growth of mutant strains and wild-type *M. smegmatis* propagated on carbon-limiting Sauton's medium containing sodium formate (A) and sodium pyruvate (B).** Growth of strains was determined by measuring the $OD_{600}$ at indicated time points and was shown on the graphs as averages from three independent experiments ± standard deviation.

**Figure S7**.

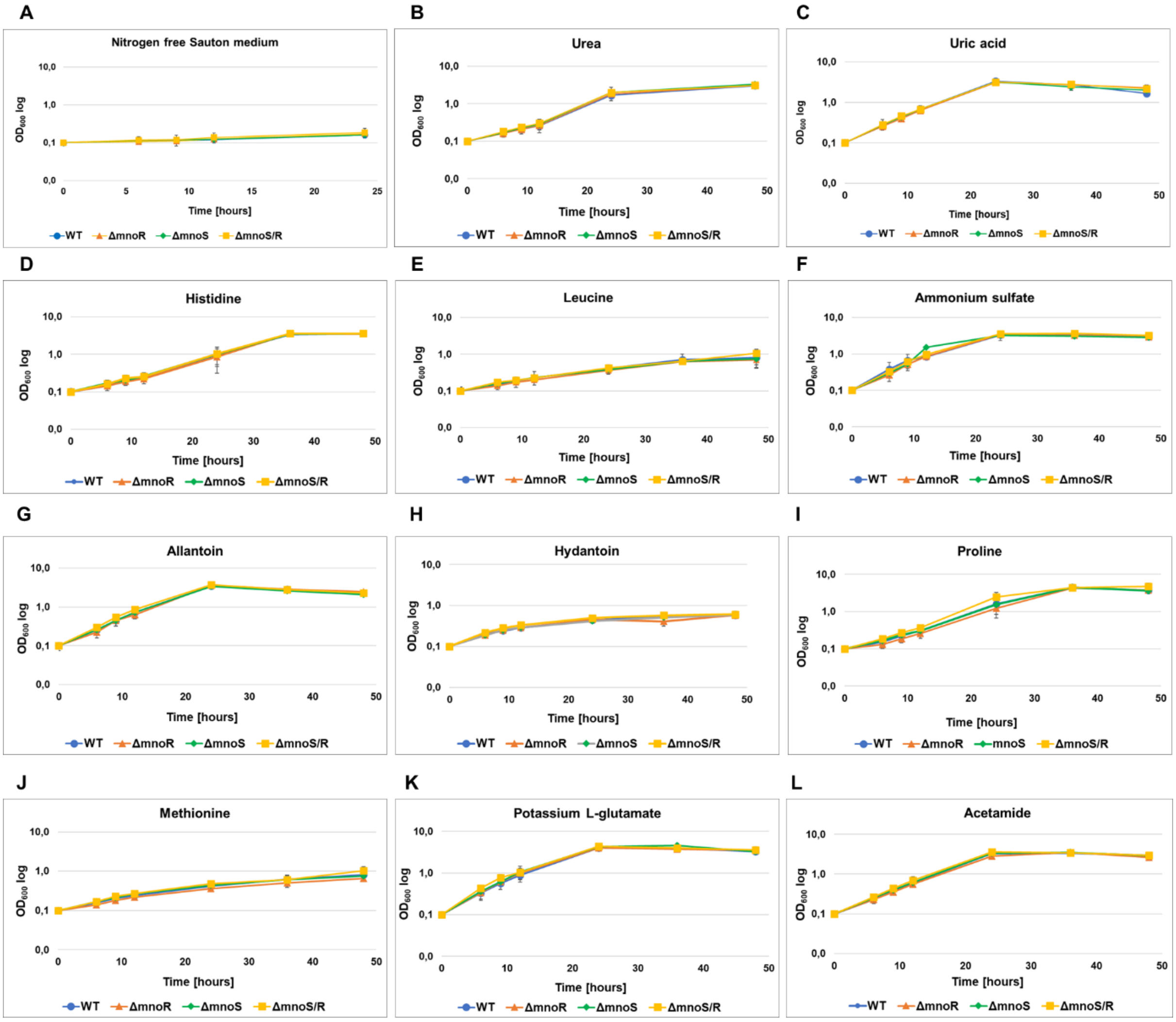


**Figure S7. Kinetics of growth of mutant strains and wild-type *M. smegmatis* propagated on nitrogen-limiting Sauton's medium containing various nitrogen sources.** Wild-type, Δ*mnoS*, Δ*mnoR*, Δ*mnoS/R* were grown in the presence of the urea (B), uric acid (C), histidine (D), leucine (E), ammonium sulfate (F), allantoin (G), hydantoin (H), proline (I), methionine (J), potassium L-glutamate (K), all at 10 mM final concentration acetamide (5mM) (L). Growth in nitrogen-free Sauton's medium was the negative control (A). Growth of strains was determined by measuring the $OD_{600}$ at indicated time points and was shown on the graphs as averages from three independent experiments ± standard deviation.

**Figure S8.**

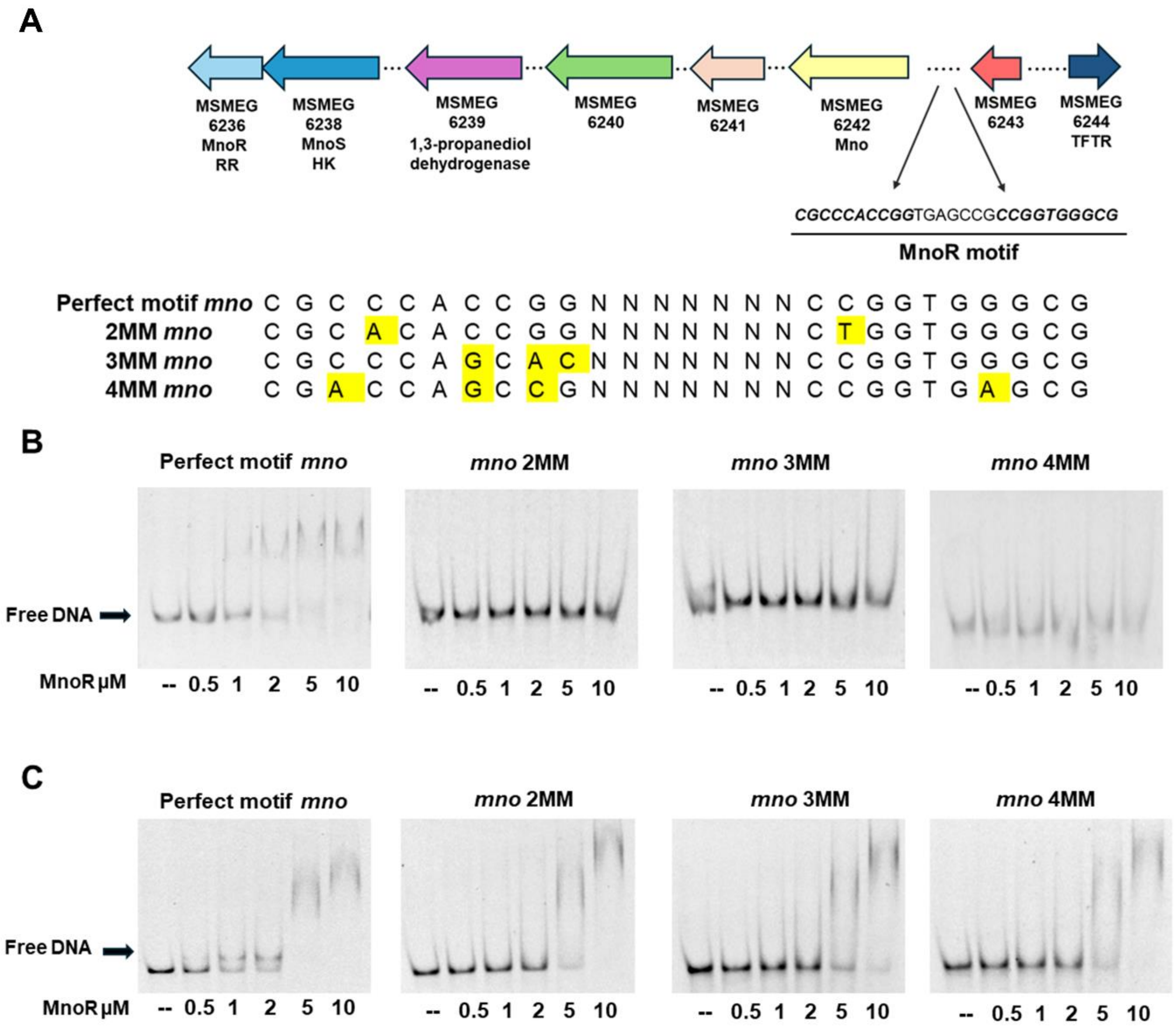


**Figure S8. Interactions between MnoR response regulator and promoter region of *msmeg_6242* (*mno*).** Perfect palindromic sequence or sequences carrying two (2MM), three (3MM), and four (4MM) mutations were analyzed by EMSA. Schematic representation of the operonic gene arrangement in the region of MnoSR two component system on the *M. smegmatis* genome. The direction of relative transcription is marked with arrows. RR – response regulator; HK – histidine kinase. Mutations in MnoR-binding sequence are marked in yellow. 5 nM of Cy5-labelled DNA was incubated with 0, 0.5, 1, 2, 5 and 10 µM MnoR in buffer containing 10 ng salmon sperm DNA **(B)** or 100:1 excess ratio of polyA DNA to Cy5-labeled oligos **(C)**. Reactions were incubated for 30 min at 25 °C, DNA-MnoR complexes were separated in 5% nondenaturing polyacrylamide gels and visualized using Azure fluorescence visualization system.

**Bibliography:**

Parish, T., & Stoker, N. G. (2000). Use of a flexible cassette method to generate a double unmarked Mycobacterium tuberculosis tlyA plcABC mutant by gene replacement. *Microbiology (Reading, England), 146 ( Pt 8)*(8), 1969–1975. https://doi.org/10.1099/00221287-146-8-1969

Sheffield, P., Garrard, S., & Derewenda, Z. (1999). Overcoming expression and purification problems of RhoGDI using a family of "parallel" expression vectors. *Protein Expression and Purification*, *15*(1), 34–39. https://doi.org/10.1006/PREP.1998.1003

Triccas, J. A., Parish, T., Britton, W. J., & Gicquel, B. (1998). An inducible expression system permitting the efficient purification of a recombinant antigen from Mycobacterium smegmatis. *FEMS Microbiology Letters*, *167*(2), 151–156. https://doi.org/10.1111/J.1574-6968.1998.TB13221.X
